# Supplementary material for: RNA-binding protein YebC enhances translation of proline-rich amino acid stretches in bacteria
Source: Nat Commun. 2025 Jul 7;16:6262. doi: 10.1038/s41467-025-60687-4 (PMC12234827; doi:10.1038/s41467-025-60687-4)
Supplement: Supplementary file 1 — Supplementary Information [file 41467_2025_60687_MOESM1_ESM.pdf]

Supplementary Information

RNA-binding protein YebC enhances translation of proline-rich amino acid stretches in bacteria

Dmitriy Ignatov[1], Vivekanandan Shanmuganathan[1†], Rina Ahmed-Begrich[1†], Kathirvel Alagesan[1], Karin Hahnke[1], Chu Wang[1], Kathrin Krause[1], Fabián A. Cornejo[1], Kristin Funke[2], Marc Erhardt[1,2], Christian Karl Frese[1], Emmanuelle Charpentier[1,2,*]

1 – Max Planck Unit for the Science of Pathogens, 10117 Berlin, Germany

2 – Institute of Biology, Humboldt-Universität zu Berlin, 10115 Berlin, Germany

* – To whom correspondence should be addressed. Tel: +49 30 28460410; Fax: +49 30 28460412; Email: research@emmanuelle-charpentier.org

† – These authors contributed equally

Present Address: Christian Karl Frese, Bayer AG, 42117 Wuppertal, Germany

Supplementary Figures

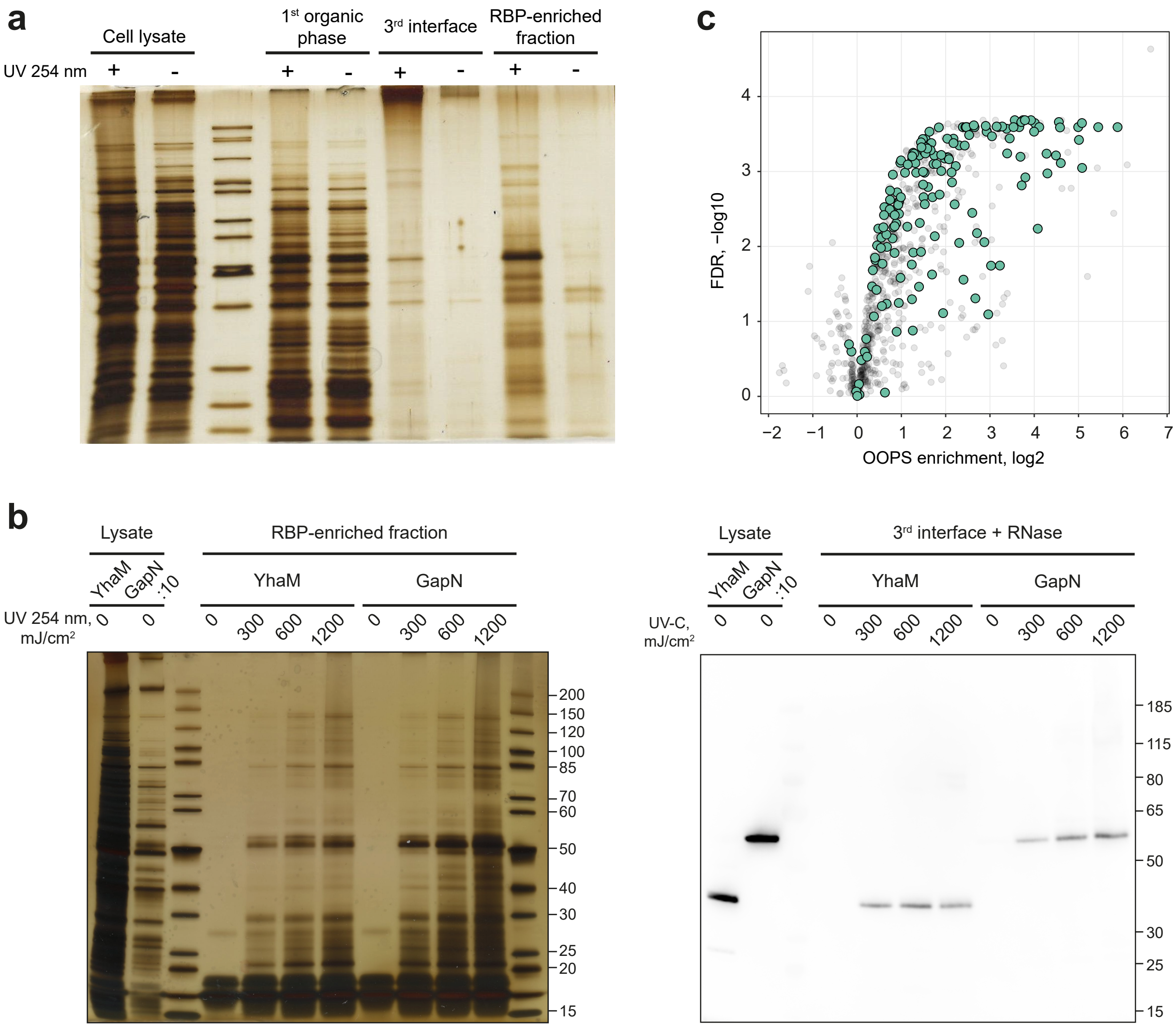

Supplementary figure 1. OOPS for *S. pyogenes*.

a UV irradiated and control samples purified according to the OOPS protocol. *S. pyogenes* cell lysates were subjected to three rounds of acid guanidinium thiocyanate-phenol-chloroform phases partition, where the interfaces between the aqueous and organic phases were collected. The third interface was treated with RNase and subjected to a new round of phases partition, where the organic phase was collected. The proteins precipitated from this organic phase are denoted as “RBP-enriched fraction”. The fractions representing the different steps of the protocol were resolved on SDS-PAGE and the gel was stained with silver. The experiment was performed one time.

b Titration of UV energy for the OOPS experiment. *S. pyogenes* strains *yhaM::3xFLAG* and *gapN::3xFLAG* were irradiated with different UV energies and RBPs were purified according to the OOPS protocol. The lysate and the RBP-enriched fraction were analysed by silver-stained SDS-PAGE and western blotting with anti-FLAG antibodies. YhaM is an annotated RNA-binding protein, while GapN is a metabolic enzyme with no demonstrated interaction with RNA. Irradiation of *S. pyogenes* cells with UV energy of 600 mJ/cm^{-2} resulted in the highest amount of YhaM relative to GapN in the RBP-enriched fraction. The experiment was performed one time.

c Volcano plot of OOPS enrichment values for *S. pyogenes* proteins. The annotated RBPs are depicted in green.

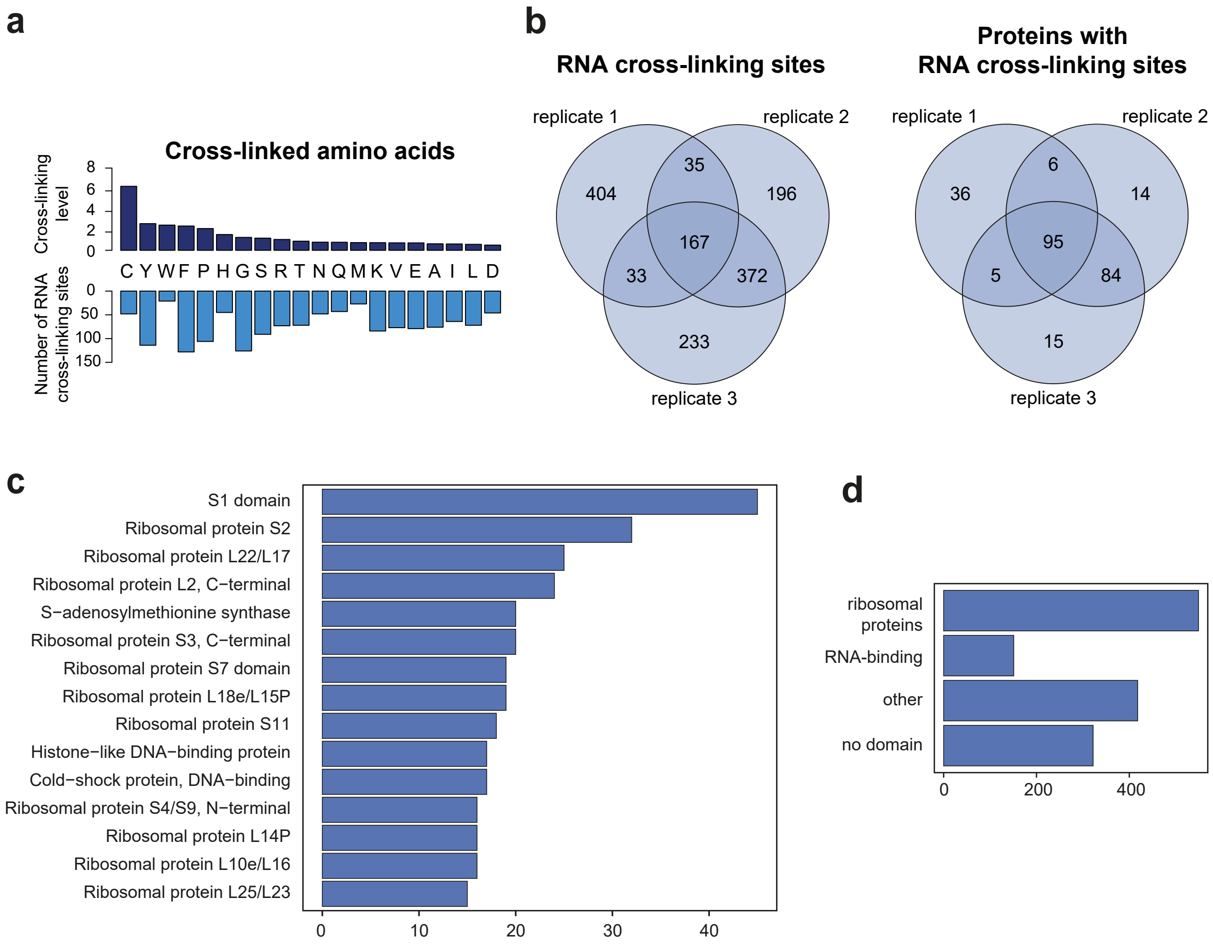

Supplementary figure 2. RBS-ID for *S. pyogenes*.

a Representation of amino acids at RNA cross-linking sites. The cross-linking levels were calculated by dividing the proportion of each amino acid in the identified RNA cross-linking sites by their proportion in the sequences of proteins where RNA cross-linking sites were identified. The corresponding counts of RNA cross-linking sites are shown below.

b Venn diagrams showing the numbers of RNA cross-linking sites and proteins with RNA cross-linking sites identified in three RBS-ID replicates and the overlap between them.

c Protein domains with the highest number of identified RNA cross-linking sites. The domains are annotated according to the PFAM database.

d Distribution of RNA cross-linking sites in domains of ribosomal proteins, RNA-binding domains, other domains and outside of annotated protein domains.

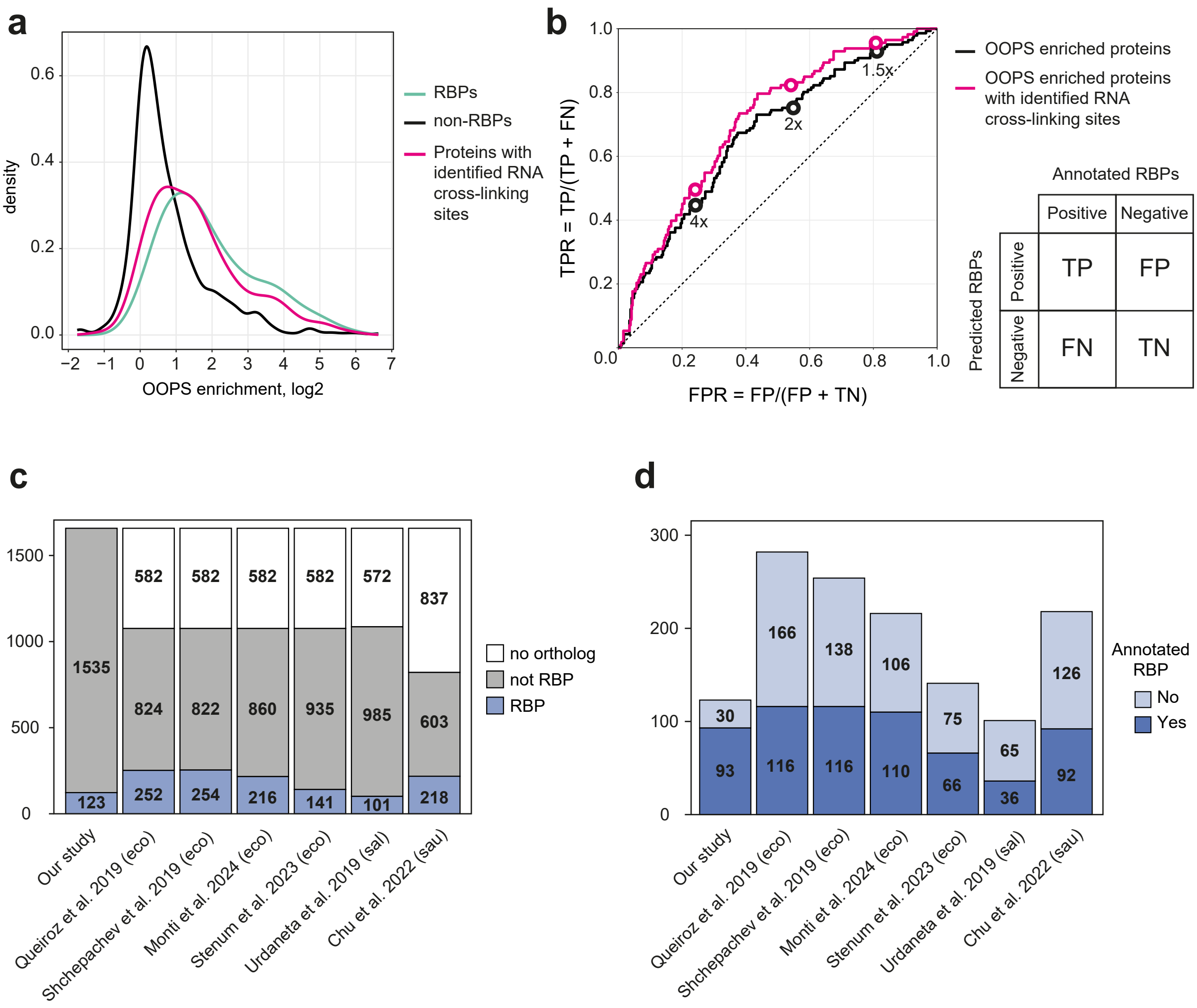

Supplementary figure 3. Identification of RBP candidates.
a Distribution of OOPS enrichment values for the annotated RBPs, non-RBPs and proteins with identified RNA cross-linking sites.
b ROC curve for the prediction of RBPs among the proteins with statistically significant OOPS enrichment. The annotated RBPs were considered true positives and all other proteins – true negatives. The OOPS enrichment values and detection of RNA cross-linking sites were used for classification. The OOPS enrichment cut-offs of 1.5, 2 and 4 are indicated on the plot.
c Orthologs of *S. pyogenes* genes in other species that have been classified as RBPs. The bars represent our study and previous studies that identified RBPs in bacteria: *E. coli* (eco), *Salmonella* Typhimurium (sal) and *Staphylococcus aureus* (sau). In our study, a protein was considered an RBP if it had a statistically significant OOPS enrichment greater than two and contained the identified RNA cross-linking sites (Supplementary Data 1). For the other studies, the number of *S. pyogenes* genes that do not have orthologs in the other bacterial species and the number of proteins that were considered RBPs are indicated.
d The number of annotated RBPs and RBP candidates among *S. pyogenes* genes whose orthologs in other species were classified as RBPs.

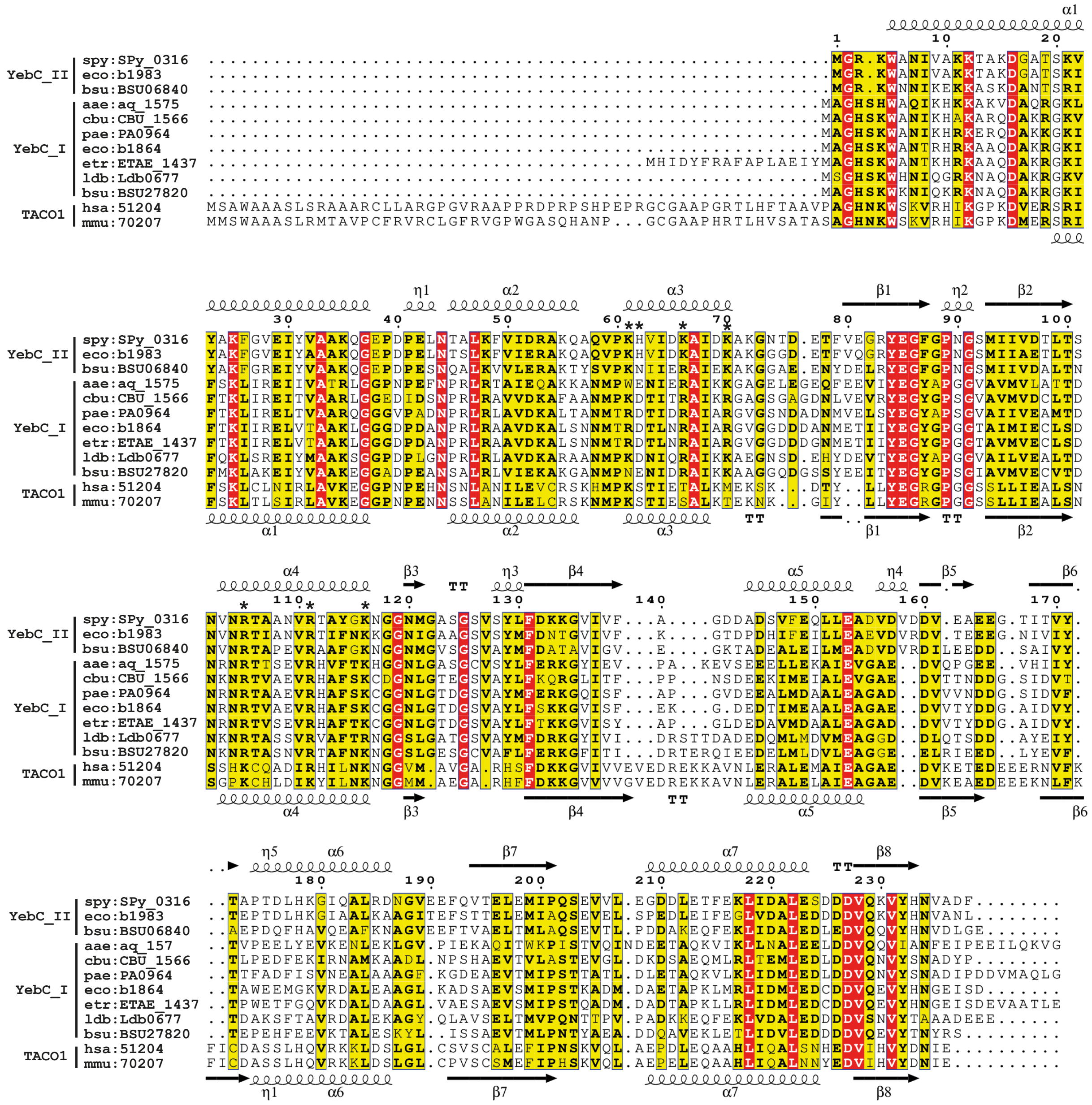

Supplementary figure 4. Alignment of YebC homologs from different organisms.
Amino acid sequences of YebC homologs from *S. pyogenes* (spy), *E. coli* (eco), *Bacillus subtilis* (bsu), *Aquifex aeolicus* (aae), *Coxiella burnetii* (cbu), *Pseudomonas aeruginosa* (pae), *Edwardsiella piscicida* (etr), *Lactobacillus delbrueckii* (ldb), *Homo sapiens* (hsa) and *Mus musculus* (mmu) were aligned with Clustal Omega. Similar residues are written with black bold characters and boxed in yellow. Strictly conserved residues are boxed in red. Secondary structures of *S. pyogenes* YebC and *M. musculus* TACO1 (PDB 5EKZ) are shown above and below the alignment respectively. The residues that were substituted with Ala in the *yebC* mutant M2 are indicated with asterisks.

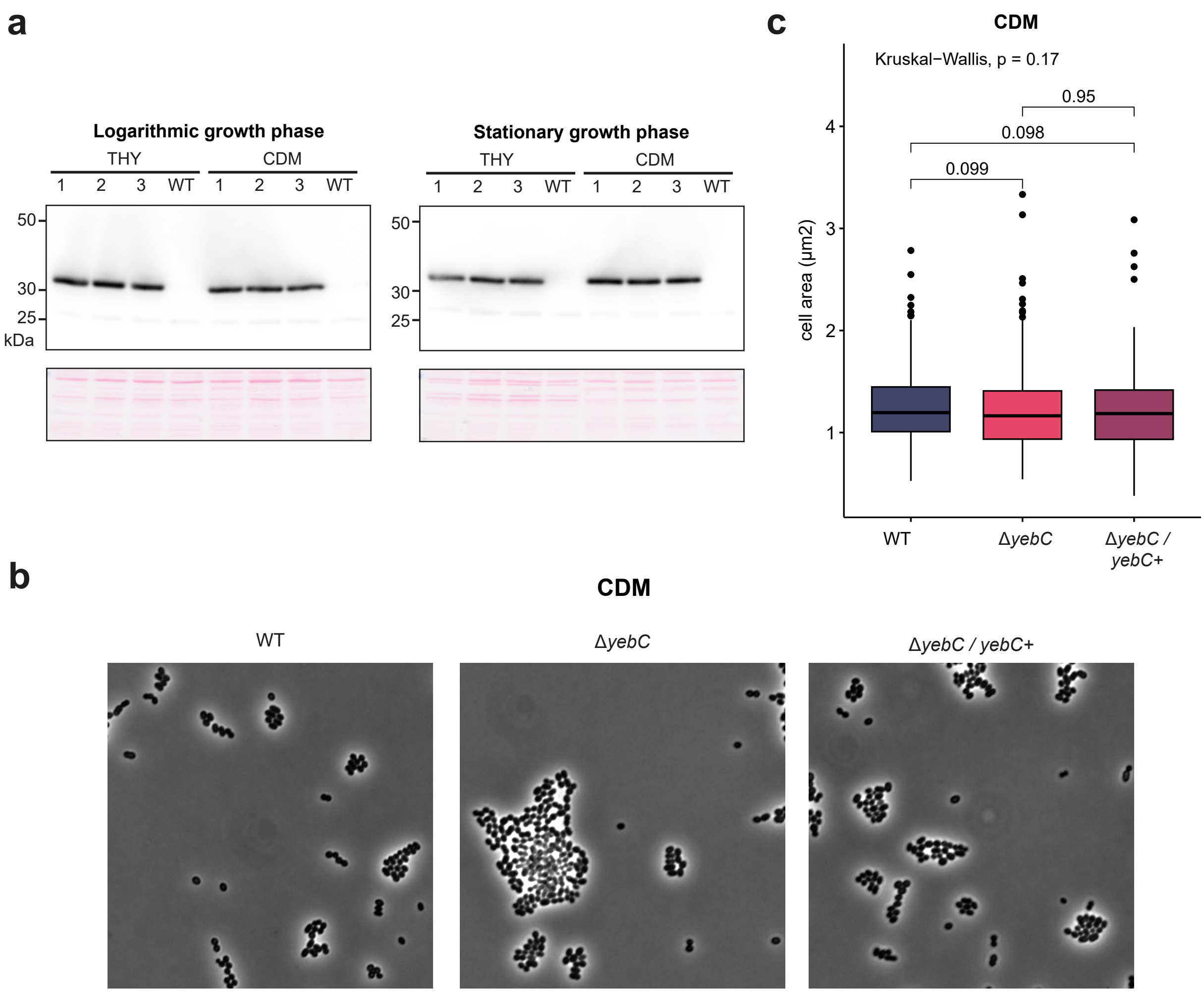

Supplementary figure 5. Analysis of *S. pyogenes* growth in CDM.

a Expression of YebC in THY and CDM. The *yebC::3xFLAG* strain was grown in the media until the logarithmic and stationary growth phases and the cells were collected and lysed. Western blotting was performed with anti-FLAG antibodies and Ponceau staining of the membrane demonstrates equal loading of samples. The cell growth and collection were performed three times, and the replicates (1-3) were analysed on the same western blot.

b Microscopy images of the overnight cultures of the WT, Δ*yebC*, and Δ*yebC / yebC*+ strains grown in CDM.

c Cell area of the overnight cultures of the WT, Δ*yebC*, and Δ*yebC / yebC*+ strains grown in CDM. The statistical difference was assessed using the Kruskal-Wallis test and unpaired Wilcoxon test. N = 3, ~ 100 cells per replicate.

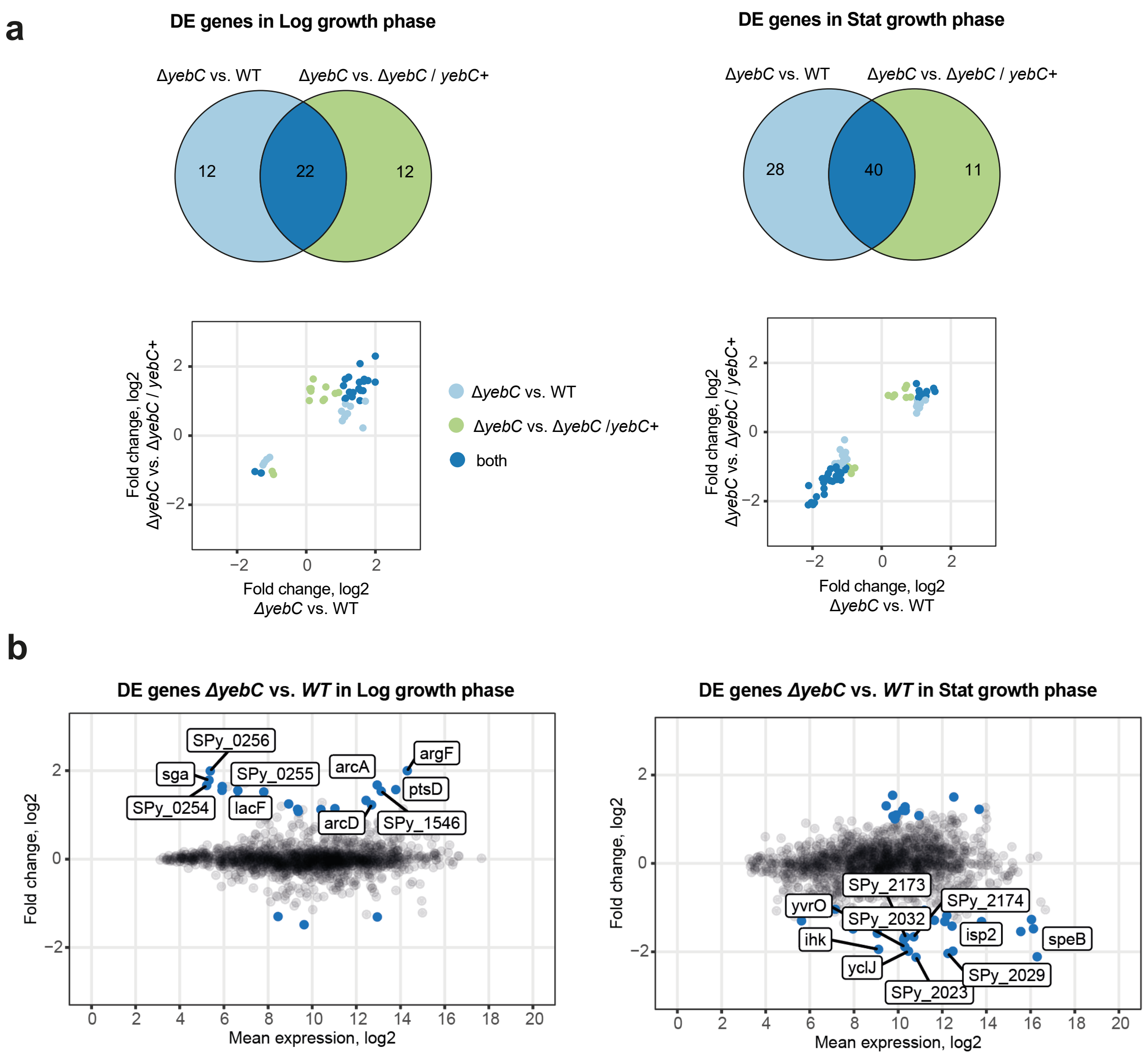

Supplementary figure 6. Transcriptome of *S. pyogenes* Δ*yebC* strain.
a Gene expression changes in the Δ*yebC* vs. WT and Δ*yebC* vs. Δ*yebC / yebC*+ strains measured in mid-logarithmic (Log) and stationary (Stat) growth phases. The Venn diagrams show the numbers of differentially expressed (DE) genes uniquely identified in one or both of the comparisons described. The scatterplots below present the fold change of the DE genes in the comparisons. The colour indicates whether the DE genes were identified only in one or in both of the comparisons.
b MA plots indicating DE genes for the Δ*yebC* vs. WT comparisons in Log and Stat growth phases. Ten DE genes with the largest fold changes are indicated.

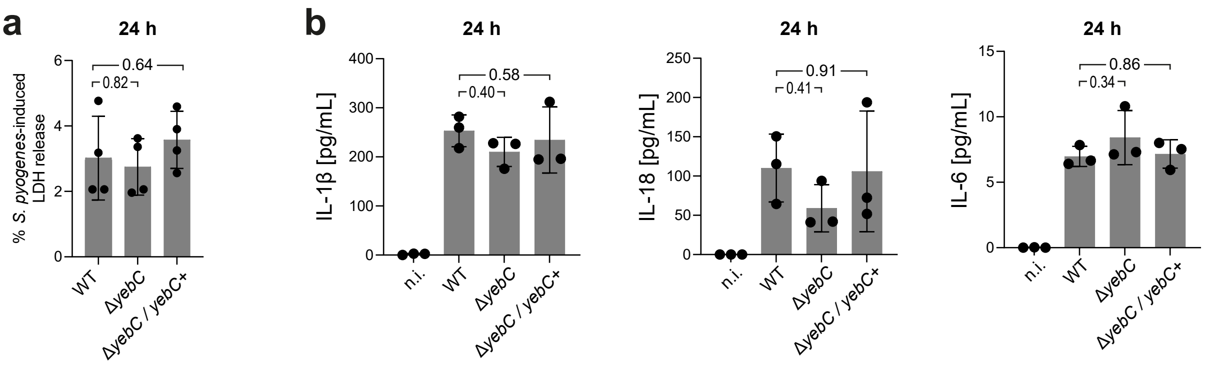

Supplementary figure 7. Infection of macrophages.
a LDH released by human macrophages infected with the WT, Δ*yebC*, and Δ*yebC / yebC+* strains at 24 h (MOI 5:1). Bars represent the mean ± standard deviation (SD) of three biological replicates. One-way ANOVA with Holm-Šídák correction for multiple comparisons was applied for statistical analyses.
b IL-1β, IL-18, and IL-6 released by human macrophages infected with the WT, Δ*yebC*, and Δ*yebC / yebC+* strains at 24 h (MOI 5:1). Bars represent the mean ± SD of three biological replicates. One-way ANOVA with Holm-Šídák correction for multiple comparisons was applied for statistical analyses.

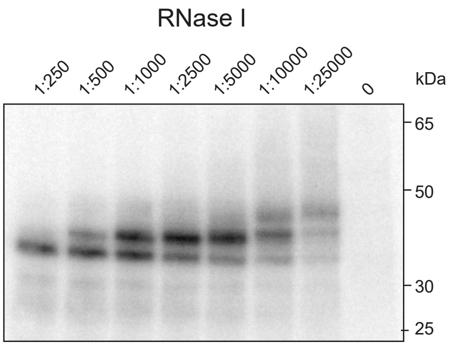

Supplementary figure 8. Migration pattern of the cross-linked YebC-RNA complexes after treatment with RNase I.
The YebC-RNA complexes cross-linked by UV irradiation were immunoprecipitated using the anti-FLAG antibodies and treated with different dilutions of RNase I. After radioactive labelling of the RNA, the complexes were resolved on the SDS-PAGE gel and transferred to the nitrocellulose membrane. The radioactive signal is not visible in the absence of RNase I suggesting that the cross-linked RNA was too long to enter the gel. The mass of the complexes increases upon RNase I dilution and at dilutions greater than 1:10000 the complexes are represented by three distinct species.

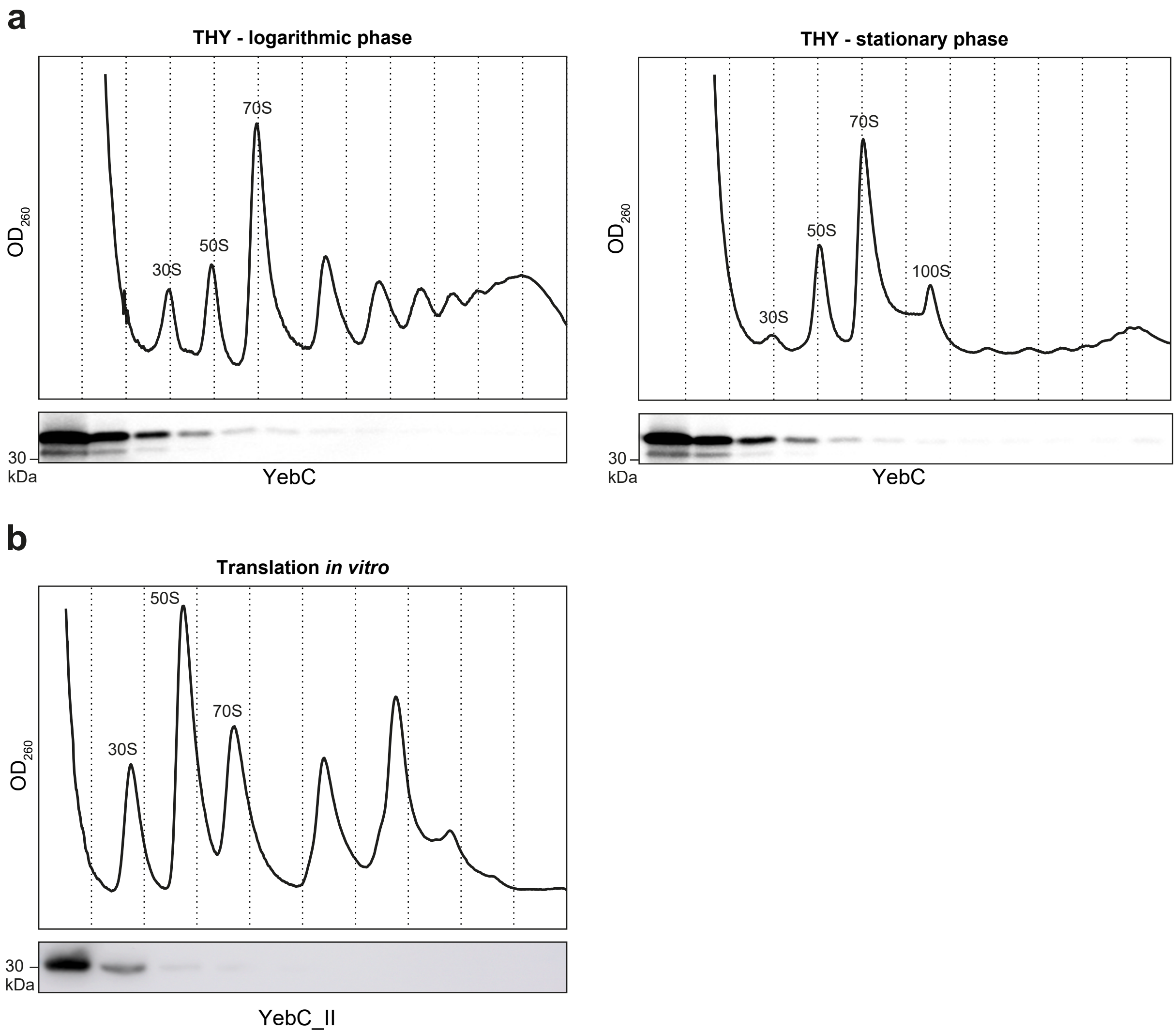

Supplementary figure 9. Association of YebC with the ribosome.
a Association of YebC with the ribosome in the logarithmic and stationary growth phases. The *yebC::3xFLAG* strain was grown in THY medium until the logarithmic and stationary growth phases and collected by rapid filtration. The lysates from each strain were resolved by sucrose density gradient ultracentrifugation. Sucrose density gradient fractions for each strain were probed with an anti-FLAG antibody. The results of the western blots are aligned with the respective $OD_{260\ nm}$ traces.
b Association of YebC_**II** (b1983) with the ribosome in the PURE *in vitro* translation system. The reaction with P5 mRNA as a template was allowed to proceed for 40 min and was resolved by ultracentrifugation in a sucrose density gradient. The sucrose density gradient fractions for each strain were probed with the anti-HA tag antibody. The result of the western blot is aligned with $OD_{260\ nm}$ trace.

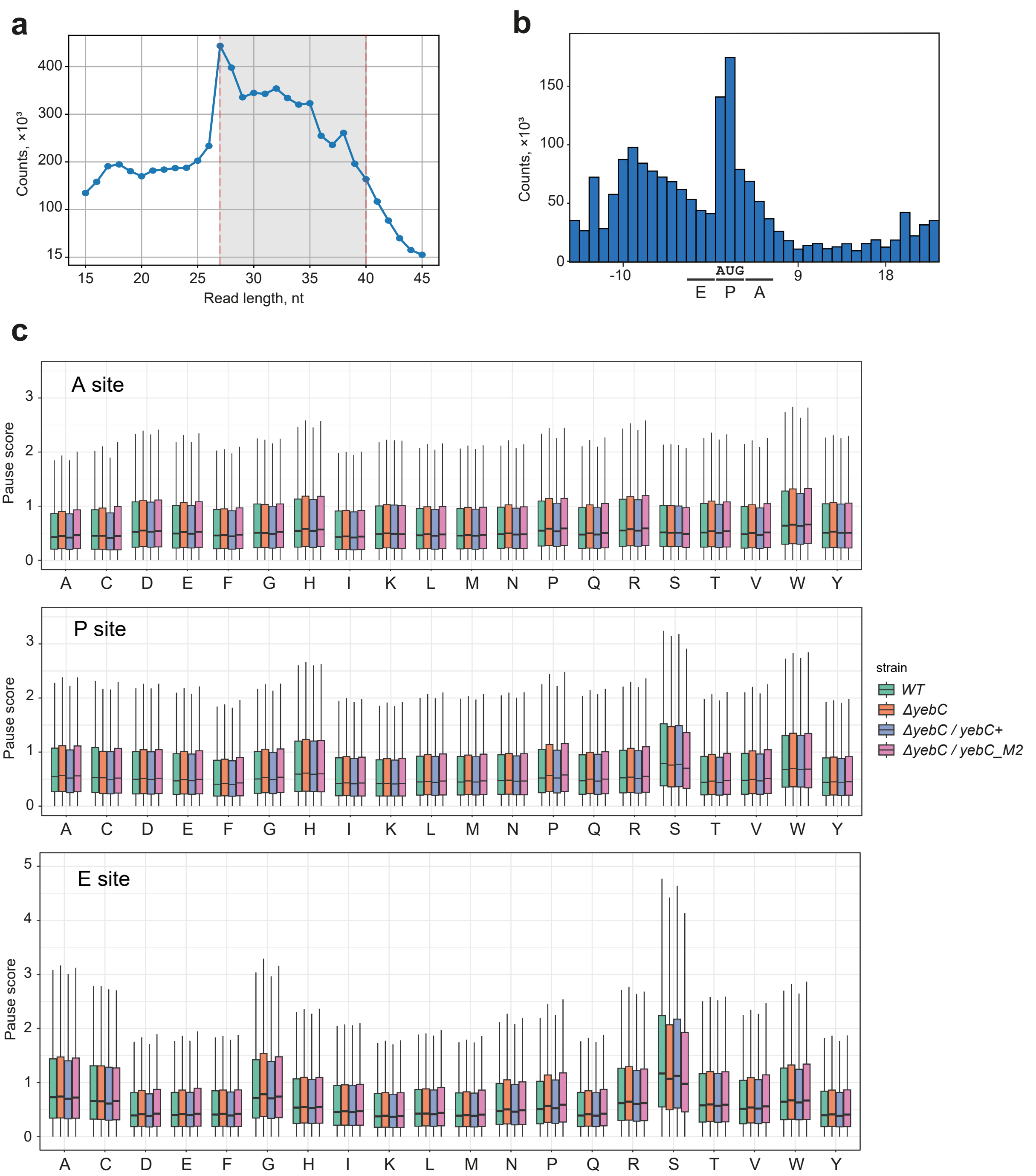

Supplementary figure 10. Analysis of the ribosome profiling results.

a Length distribution of mapped reads in the WT replicate 1 sample. The reads having lengths between 27 and 40 nucleotides were selected for further analysis.

b Assignment of the ribosome P site position near the start codon. The P site position for each mapped cDNA read was determined by shifting the 3′ end of the read by 15 nucleotides. The sum of the P site positions near the start codon in WT replicate 1 is presented. The increased number of P sites assigned to the start codon indicates the correct assignment of the ribosome position.

c Distribution of the pause scores for the codons in the A, P and E sites of the ribosome in the WT and *yebC* mutants. For each codon, the average pause score of three replicates was calculated in each sample. The distribution of the mean pause scores is represented by a boxplot, where the box represents the interquartile range and the median and the whiskers extend up to ×1.5 of the interquartile range.

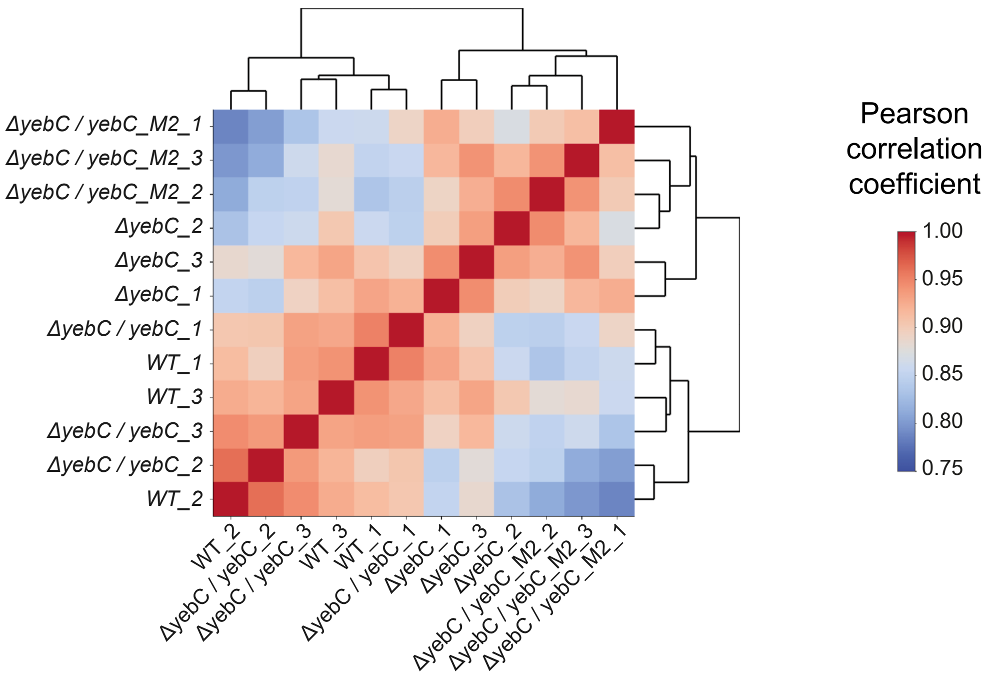

Supplementary figure 11. Correlation between the ribosome profiling samples.
The Pearson correlation coefficients were calculated between the pause scores of the ribosome profiling samples. The samples with high correlation coefficients are clustered together.

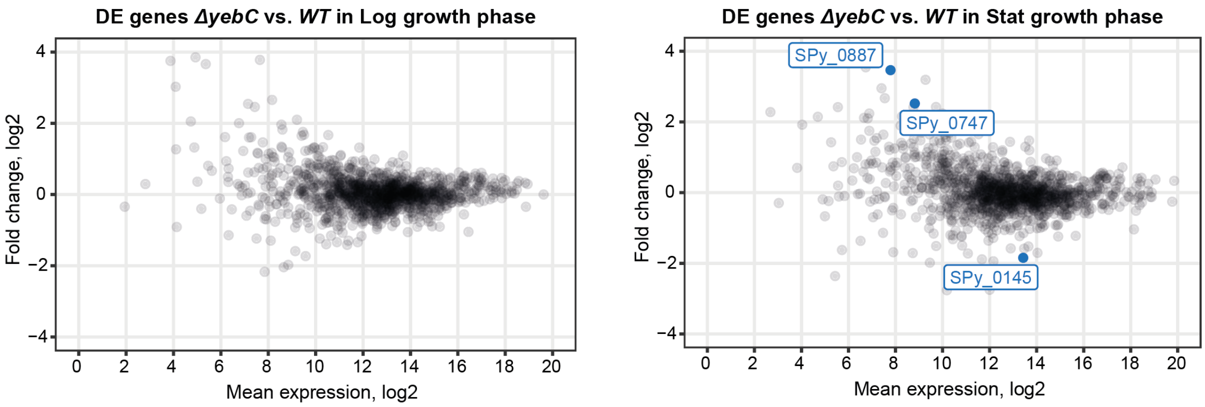

Supplementary figure 12. MS proteomics of the *yebC* mutant.
MA plots indicating DE genes for the Δ*yebC* vs. WT comparisons in the logarithmic and stationary growth phases. The genes with statistically significant differential expression are indicated.

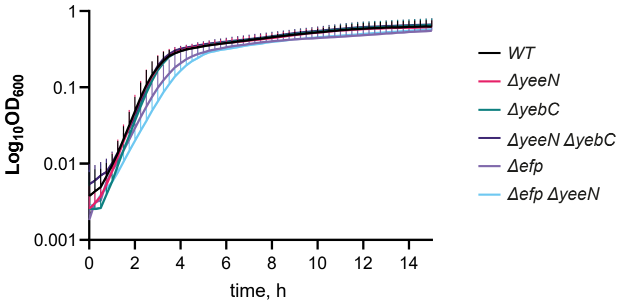

Supplementary figure 13. Growth curves of *Salmonella* Typhimurium mutants.
The indicated strains were grown in LB medium and the growth curves were plotted. The experiment was performed six times independently, and the mean + SD of $OD_{600\ nm}$ values are presented.

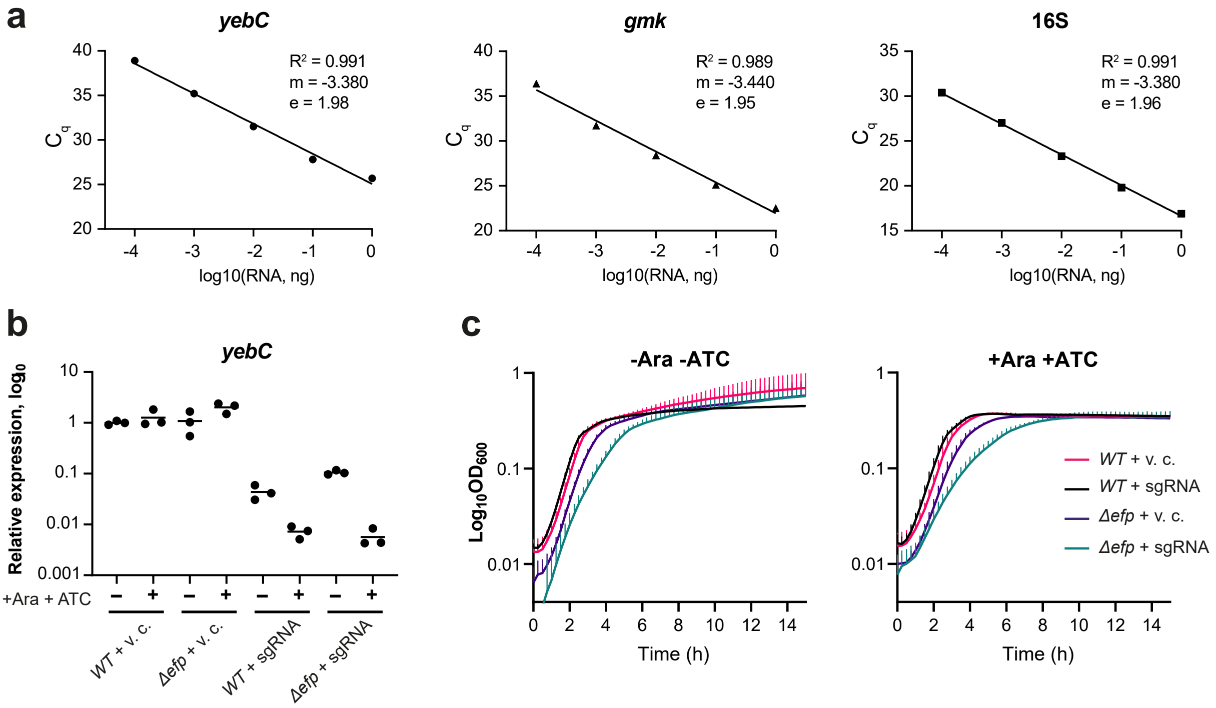

Supplementary figure 14. Depletion of *yebC* in *Salmonella* Typhimurium mutants using CRISPRi.
a Calculation of qRT-PCR primer efficiencies. Serial dilutions of *S.* Typhimurium RNA served as an input for qRT-PCR with the indicated primers. C_q values were plotted against the log10-transformed RNA input, and the coefficient of determination (R^2), slope of the regression line (m) and primer efficiency (e) were calculated for each primer pair. The experiment was performed once with technical duplicates.
b Relative expression of *yebC* mRNA measured by qRT-PCR. *S.* Typhimurium WT and Δ*efp* strains were transformed with pdCas9-sgRNA-RFP (vector control) or pdCas9-sgRNA-yebC (sgRNA). The cells were grown in LB supplemented with 10 µg/mL chloramphenicol with or without inducers (10 mM arabinose and 100 ng/mL anhydrotetracycline) until the mid-logarithmic growth phase. The abundance of *yebC* mRNA was measured by qRT-PCR with *gmk* and 16S rRNA as reference genes. The bands represent the means of three independent biological replicates.
c Growth curves of strains described in panel B. The mean + SD of six biological replicates are presented.

pEC2817 (pSEVA141_lox71-*erm*-lox66)

lox71 lox66 ermAMB

<pSEVA141_backbone>GAATTCGAGCTCGGTACCCTACCGTTCGTATAGCATACATTATACGAAGTTATCCGTAGCGGTTTTCAAAATTTG
CAACCAGGAATGAATTACTATCCCTTTTATCAAGAAGCGCAAAAGAAAAACGAAATGATACACCAATCAGTGCAAAAAAAGATATAATGGGAGA
TAAGACGGTTCGTGTTCGTGCTGACTTGCACCATATCATAAAAATCGAAACAGCAAAGAATGGCGGAAACGTAAAAGAAGTTATGGAAATAAGA
CTTAGAAGCAAACTTAAGAGTGTGTTGATAGTGCAGTATCTTAAAATTTTGTATAATAGGAATTGAAGTTAAATTAGATGCTAAAAATTTGTAA
TTAAGAAGGAGTGATTACATGAACAAAAATATAAAATATTCTCAAAACTTTTTAACGAGTGAAAAAGTACTCAACCAAATAATAAAACAATTGA
ATTTAAAAGAAACCGATACCGTTTACGAAATTGGAACAGGTAAAGGGCATTTAACGACGAAACTGGCTAAAATAAGTAAACAGGTAACGTCTAT
TGAATTAGACAGTCATCTATTCAACTTATCGTCAGAAAAATTAAAACTGAATACTCGTGTCACTTTAATTCACCAAGATATTCTACAGTTTCAA
TTCCCTAACAAACAGAGGTATAAAATTGTTGGGAGTATTCCTTACCATTTAAGCACACAAATTATTAAAAAAGTGGTTTTTGAAAGCCATGCGT
CTGACATCTATCTGATTGTTGAAGAAGGATTCTACAAGCGTACCTTGGATATTCACCGAACACTAGGGTTGCTCTTGCACACTCAAGTCTCGAT
TCAGCAATTGCTTAAGCTGCCAGCGGAATGCTTTCATCCTAAACCAAAAGTAAACAGTGTCTTAATAAAACTTACCCGCCATACCACAGATGTT
CCAGATAAATATTGGAAGCTATATACGTACTTTGTTTCAAAATGGGTCAATCGAGAATATCGTCAACTGTTTACTAAAAATCAGTTTCATCAAG
CAATGAAACACGCCAAAGTAAACAATTTAAGTACCGTTACTTATGAGCAAGTATTGTCTATTTTTAATAGTTATCTATTATTTAACGGGAGGAA
ATAAATAACTTCGTATAGCATACATTATACGAACGGTAGGGGATCCTCTAGAGTCGACCTGCAGGCATGCAAGCTT<pSEVA141_backbone
>

pEC2818 (pSEVA141_3xFLAG-lox71-*erm*-lox66)

C-terminal 3xFLAG lox71 lox66 ermAMB

<pSEVA141_backbone>GAATTCCCATGGGAGCTCGGTACCGACTACAAAGACCATGACGGTGATTATAAAGATCATGATATCGACTACAAA
GATGACGACGATAAATAGTAAGTACCCTACCGTTCGTATAGCATACATTATACGAAGTTATCCGTAGCGGTTTTCAAAATTTGCAACCAGGAAT
GAATTACTATCCCTTTTATCAAGAAGCGCAAAAGAAAAACGAAATGATACACCAATCAGTGCAAAAAAAGATATAATGGGAGATAAGACGGTTC
GTGTTCGTGCTGACTTGCACCATATCATAAAAATCGAAACAGCAAAGAATGGCGGAAACGTAAAAGAAGTTATGGAAATAAGACTTAGAAGCAA
ACTTAAGAGTGTGTTGATAGTGCAGTATCTTAAAATTTTGTATAATAGGAATTGAAGTTAAATTAGATGCTAAAAATTTGTAATTAAGAAGGAG
TGATTACATGAACAAAAATATAAAATATTCTCAAAACTTTTTAACGAGTGAAAAAGTACTCAACCAAATAATAAAACAATTGAATTTAAAAGAA
ACCGATACCGTTTACGAAATTGGAACAGGTAAAGGGCATTTAACGACGAAACTGGCTAAAATAAGTAAACAGGTAACGTCTATTGAATTAGACA
GTCATCTATTCAACTTATCGTCAGAAAAATTAAAACTGAATACTCGTGTCACTTTAATTCACCAAGATATTCTACAGTTTCAATTCCCTAACAA
ACAGAGGTATAAAATTGTTGGGAGTATTCCTTACCATTTAAGCACACAAATTATTAAAAAAGTGGTTTTTGAAAGCCATGCGTCTGACATCTAT
CTGATTGTTGAAGAAGGATTCTACAAGCGTACCTTGGATATTCACCGAACACTAGGGTTGCTCTTGCACACTCAAGTCTCGATTCAGCAATTGC
TTAAGCTGCCAGCGGAATGCTTTCATCCTAAACCAAAAGTAAACAGTGTCTTAATAAAACTTACCCGCCATACCACAGATGTTCCAGATAAATA
TTGGAAGCTATATACGTACTTTGTTTCAAAATGGGTCAATCGAGAATATCGTCAACTGTTTACTAAAAATCAGTTTCATCAAGCAATGAAACAC
GCCAAAGTAAACAATTTAAGTACCGTTACTTATGAGCAAGTATTGTCTATTTTTAATAGTTATCTATTATTTAACGGGAGGAAATAAATAACTT
CGTATAGCATACATTATACGAACGGTAGGGGATCCTCTAGAGTCGACCTGCAGGCATGCAAGCTT<pSEVA141_backbone>

Supplementary figure 15. Nucleotide sequences introduced to the pSEVA141 backbone. The partial sequences of the plasmids pSEVA141-lox71-erm-lox66 and pSEVA141-3xFLAG-lox71-erm-lox66 are presented. The coding sequences and other functional elements are indicated with colours.

pEC3183 (p7INTΔlacZα_Ptet_sfGFP)

tetR tetO sfGFP

<p7INT_backbone>CCAGCTGAATTCAAAAAAGGCCCACTTTTGTGGGCCTTTTTTTTAAGACCCACTTTCACATTTAAGTTGTTTTTCTAA
TCCGCAGATGATCAATTCAAGGCCGAATAAGAAGGCTGGCTCTGCACCTTGGTGATCAAATAATTCGATAGCTTGTCGTAATAATGGCGGCATA
CTATCAGTAGTAGGTGTTTCCCTTTCTTCTTTAGCGACTTGATGCTCTTGATCTTCCAATACGCAACCTAAAGTAAAATGCCCCACAGCGCTGA
GTGCATATAATGCATTCTCTAGTGAAAAACCTTGTTGGCATAAAAAGGCTAATTGATTTTCGAGAGTTTCATACTGTTTTTCTGTAGGCCGTGT
ACCTAAATGTACTTTTGCTCCATCGCGATGACTTAGTAAAGCACATCTAAAACTTTTAGCGTTATTACGTAAAAAATCTTGCCAGCTTTCCCCT
TCTAAAGGGCAAAAGTGAGTATGGTGCCTATCTAACATCTCAATGGCTAAGGCGTCGAGCAAAGCCCGCTTATTTTTTACATGCCAATACAATG
TAGGCTGCTCTACACCTAGCTTCTGGGCGAGTTTACGGGTTGTTAAACCTTCGATTCCGACCTCATTAAGCAGCTCTAATGCGCTGTTAATCAC
TTTACTTTTATCTAATCTAGACATCATTAATTCCTCCTTTTTGTTGACACTCTATCATTGATAGAGTTATTTGTCAAACTAGTTTTTTATTTGG
ATCCCCTCGAGTTCATGAAAAACTAAAAAAAATATTGACACTCTATCATTGATAGAGTATAATTAAAATAAGACTCTATCATTGATAGAGTCTT
GATGGTACCGAGCTCGAATAGATCTTCGAGTCTAGTTAAGGAGGTGATCTCATATGAGCAAAGGAGAAGAACTTTTCACTGGAGTTGTCCCAAT
TCTTGTTGAATTAGATGGTGATGTTAATGGGCACAAATTTTCTGTCCGTGGAGAGGGTGAAGGTGATGCTACAAACGGAAAGCTTACCCTTAAA
TTTATTTGCACTACTGGAAAACTACCTGTTCCATGGCCAACACTTGTCACTACTCTCACTTATGGTGTTCAATGCTTTTCCCGTTATCCGGATC
ATATGAAACGGCATGACTTTTTCAAGAGTGCCATGCCCGAAGGTTATGTACAGGAACGCACTATATCTTTCAAAGATGACGGGACTTACAAGAC
GCGTGCTGAAGTCAAGTTTGAAGGTGATACCCTTGTTAATCGTATCGAGTTAAAAGGTATTGATTTTAAAGAAGATGGAAACATTCTCGGACAC
AAACTTGAGTACAACTTTAACTCACACAATGTATACATCACGGCAGACAAACAAAAGAATGGAATCAAAGCTAACTTCAAAATTCGCCACAACG
TTGAAGATGGATCCGTTCAACTAGCAGACCATTATCAACAAAATACTCCAATTGGCGATGGCCCTGTCCTTTTACCAGACAACCATTACCTGTC
GACACAATCTGTCCTTTCGAAAGATCCCAACGAAAAGCGTGACCACATGGTCCTTCTTGAGTTTGTAACTGCTGCTGGGATTACACATGGCATG
GATGAGCTCTACAAATAATGGCTAAAAGCGACAGAAAAACTGAAATTTTTCTGTTTTTCTGTTAAAATAGGGTCTAGACGTAATAGCGAAGAGG
C<p7INT_backbone>

pEC3160 (p7INTΔlacZα_Ptet_3xFLAG-sfGFP-P0-mKate)

tetR tetO 3xFLAG sfGFP mKate2

<p7INT_backbone>CCAGCTGAATTCAAAAAAGGCCCACTTTTGTGGGCCTTTTTTTTAAGACCCACTTTCACATTTAAGTTGTTTTTCTAA
TCCGCAGATGATCAATTCAAGGCCGAATAAGAAGGCTGGCTCTGCACCTTGGTGATCAAATAATTCGATAGCTTGTCGTAATAATGGCGGCATA
CTATCAGTAGTAGGTGTTTCCCTTTCTTCTTTAGCGACTTGATGCTCTTGATCTTCCAATACGCAACCTAAAGTAAAATGCCCCACAGCGCTGA
GTGCATATAATGCATTCTCTAGTGAAAAACCTTGTTGGCATAAAAAGGCTAATTGATTTTCGAGAGTTTCATACTGTTTTTCTGTAGGCCGTGT
ACCTAAATGTACTTTTGCTCCATCGCGATGACTTAGTAAAGCACATCTAAAACTTTTAGCGTTATTACGTAAAAAATCTTGCCAGCTTTCCCCT
TCTAAAGGGCAAAAGTGAGTATGGTGCCTATCTAACATCTCAATGGCTAAGGCGTCGAGCAAAGCCCGCTTATTTTTTACATGCCAATACAATG
TAGGCTGCTCTACACCTAGCTTCTGGGCGAGTTTACGGGTTGTTAAACCTTCGATTCCGACCTCATTAAGCAGCTCTAATGCGCTGTTAATCAC
TTTACTTTTATCTAATCTAGACATCATTAATTCCTCCTTTTTGTTGACACTCTATCATTGATAGAGTTATTTGTCAAACTAGTTTTTTATTTGG
ATCCCCTCGAGTTCATGAAAAACTAAAAAAAATATTGACACTCTATCATTGATAGAGTATAATTAAAATAAGACTCTATCATTGATAGAGTCTT
GATGGTACCGAGCTCGAATAGATCTTCGAGTCTAGTTAAGGAGGTGATCTCATATGGACTACAAGGATCATGATGGTGATTATAAAGATCATGA
TATCGATTACAAAGACGATGACGACAAGAGCAAAGGAGAAGAACTTTTCACTGGAGTTGTCCCAATTCTTGTTGAATTAGATGGTGATGTTAAT
GGGCACAAATTTTCTGTCCGTGGAGAGGGTGAAGGTGATGCTACAAACGGAAAGCTTACCCTTAAATTTATTTGCACTACTGGAAAACTACCTG
TTCCATGGCCAACACTTGTCACTACTCTCACTTATGGTGTTCAATGCTTTTCCCGTTATCCGGATCATATGAAACGGCATGACTTTTTCAAGAG
TGCCATGCCCGAAGGTTATGTACAGGAACGCACTATATCTTTCAAAGATGACGGGACTTACAAGACGCGTGCTGAAGTCAAGTTTGAAGGTGAT
ACCCTTGTTAATCGTATCGAGTTAAAAGGTATTGATTTTAAAGAAGATGGAAACATTCTCGGACACAAACTTGAGTACAACTTTAACTCACACA
ATGTATACATCACGGCAGACAAACAAAAGAATGGAATCAAAGCTAACTTCAAAATTCGCCACAACGTTGAAGATGGATCCGTTCAACTAGCAGA
CCATTATCAACAAAATACTCCAATTGGCGATGGCCCTGTCCTTTTACCAGACAACCATTACCTGTCGACACAATCTGTCCTTTCGAAAGATCCC
AACGAAAAGCGTGACCACATGGTCCTTCTTGAGTTTGTAACTGCTGCTGGGATTACACATGGCATGGATGAGCTCTACAAAGGATCTGTTTCAG
AACTTATCAAAGAAAACATGCACATGAAACTTTACATGGAAGGTACTGTTAACAACCACCACTTCAAATGTACTTCAGAAGGTGAAGGTAAACC
ATACGAAGGTACTCAAACTATGCGTATCAAAGCTGTTGAAGGTGGTCCACTTCCATTCGCTTTCGACATCCTTGCTACTTCATTCATGTACGGT
TCAAAAACTTTCATCAACCACACTCAAGGTATCCCAGACTTCTTCAAACAATCATTCCCAGAAGGTTTCACTTGGGAACGTGTTACTACTTACG
AAGACGGTGGTGTTCTTACTGCTACTCAAGACACTTCACTTCAAGACGGTTGTCTTATCTACAACGTTAAAATCCGTGGTGTTAACTTCCCATC
AAACGGTCCAGTTATGCAAAAAAAAACTCTTGGTTGGGAAGCTTCAACTGAAACTCTTTACCCAGCTGACGGTGGTCTTGAAGGTCGTGCTGAC
ATGGCTCTTAAACTTGTTGGTGGTGGTCACCTTATCTGTAACCTTAAAACTACTTACCGTTCAAAAAAACCAGCTAAAAACCTTAAAATGCCAG
GTGTTTACTACGTTGACCGTCGTCTTGAACGTATCAAAGAAGCTGACAAAGAAACTTACGTTGAACAACACGAAGTTGCTGTTGCTCGTTACTG
TGACCTTCCATCAAAACTTGGTCACCGTGCATAATAGTGACTGACGAAAAAAGGCCCACTTTTGTGGGCCTTTTTT<p7INT_backbone>

Supplementary figure 16. Nucleotide sequences introduced to the p7INT backbone.
The partial sequences of the plasmids p7INTΔlacZα_P*tet*_sfGFP and p7INTΔlacZα_ P*tet*_3xFLAG-sfGFP-P0-mKate are presented. The coding sequences and other functional elements are indicated with colours.

Supplementary Tables

Supplementary Table 1: Primers used in the study

Code	Name	Sequence
**Introduction of 3x FLAG tag to the C-termini of *S. pyogenes* genes**		
OLEC11928	3FLAG_yhaM_up_EcoRI_F	GATCAGAATTCCCTCAGGTTAATCAAATCACC
OLEC11929	3FLAG_yhaM_up_KpnI_R	GATCAGGTACCATAGTTTGGCTTGTAAAAAGACC
OLEC11930	3FLAG_yhaM_dw_BamHI_F	GATCAGGATCCTCAGTGTTTCTCGAGTAATAGTTC
OLEC11931	3FLAG_yhaM_dw_SalI_R	GATCAGTCGACCTACGCTTTGACAGAAACATC
OLEC11932	3FLAG_yhaM_seq_F	GGGCAATTAGAATACGGTAGTC
OLEC11933	3FLAG_yhaM_seq_R	TTGTTCTTATTAATATCATGCAAGAATTC
OLEC11934	3FLAG_gapN_up_EcoRI_F	GATCAGAATTCTTAAACCACCAACACAAGGCT
OLEC11935	3FLAG_gapN_up_KpnI_R	GATCAGGTACCCTGGATATCAAATACAACAGATTTAAC
OLEC11936	3FLAG_gapN_dw_BamHI_F	GATCAGGATCCTAAAAAAAATAAACAAAAAGTTAGGTTAAC
OLEC11937	3FLAG_gapN_dw_PstI_R	GATCACTGCAGGCCAAAATATCTAAAACTTGAGGA
OLEC11938	3FLAG_gapN_seq_F	GCATTGCTGAGCAATTAGAAG
OLEC11939	3FLAG_gapN_seq_R	AAACTATACGCTGACTATCTTCG
OLEC12918	3FLAG_yebC_up_NcoI_F	ACACACCATGGGTAAATGGGCAAATATTGTTGC
OLEC12919	3FLAG_yebC_up_KpnI_R	ACACAGGTACCAAAATCTGCTACATTATGATACAC
OLEC12920	3FLAG_yebC_dw_BamHI_F	ACACAGGATCCATTGACATAGAATAATAAAAAGAGTTG
OLEC12921	3FLAG_yebC_dw_PstI_R	ACACACTGCAGGCTCTGTTGAGGTGAGTTC
OLEC12922	3FLAG_yebC_seq_F	AAGTGCTATAATAAGGGAGTTAG
OLEC12923	3FLAG_ko_yebC_seq_R	TCCATCAGCAGTCAACTC
OLEC13855	3FLAG_phoH_up_NcoI_F	ACACACCATGGGCTTTATCTATGGCAGAATCTC
OLEC13856	3FLAG_phoH_up_KpnI_R	ACACAGGTACCGTGCTCTTGACCGATCAC
OLEC13857	3FLAG_phoH_dw_XbaI_F	ACACATCTAGAGACTTTTACGATGATGTTTAATATGG
OLEC13858	3FLAG_phoH_dw_PaeI_R	ACACAGCATGCCCCTTATCCTTTCAATCAGC
OLEC13941	3FLAG_phoH_seq_F	GTGATGATGAAGAAGCTG
OLEC13942	3FLAG_phoH_seq_R	AATCATCTCGATATACATAAGG
OLEC13859	3FLAG_thuC_up_NcoI_F	ACACACCATGGTTTCTTTTGATGTGCCTGAC
OLEC13860	3FLAG_thuC_up_KpnI_R	ACACAGGTACCTGAGTTTTTCTTAACATTTTCTATAATCG
OLEC13861	3FLAG_thuC_dw_BamHI_F	ACACAGGATCCTGTGGGTGAATTTGGGG
OLEC13862	3FLAG_thuC_dw_SalI_R	ACACAGTCGACTCGATATCATTGCTGGATAG
OLEC13943	3FLAG_thuC_seq_F	AATTTAATCTTGTCAACTCAAC
OLEC13944	3FLAG_thuC_seq_R	CATTCTACCTCTGACAGC
OLEC12950	3FLAG_yjbK_up_NcoI_F	ACACACCATGGGTCTCCTTTCTTGTCAAAGC
OLEC12951	3FLAG_yjbK_up_KpnI_R	ACACAGGTACCTTTATCGTTGAACTTTTTTAAGGTA
OLEC12952	3FLAG_yjbK_dw_BamHI_F	ACACAGGATCCTGGCTAAAAGCGACAGAAAAAC
OLEC12953	3FLAG_yjbK_dw_PstI_R	ACACACTGCAGTTTAGCAGCTTCTGCAAAGG
OLEC12954	3FLAG_yjbK_seq_F	CGATATTGCTTGCGAATACC

OLEC12955	3FLAG_yjbK_seq_R	CTCCACCAGCAAATAAGC
OLEC12966	3FLAG_ygaC_up_NcoI_F	ACACACCATGGGCGACGACATTAATTGCAAG
OLEC12967	3FLAG_ygaC_up_KpnI_R	ACACAGGTACCACGATTCTTCAGTTCAAGATAAC
OLEC12968	3FLAG_ygaC_dw_BamHI_F	ACACAGGATCCGAGTTGTCCAGCACTCC
OLEC12969	3FLAG_ygaC_dw_PstI_R	ACACACTGCAGTTTGTCTGTTTTACGAACGTG
OLEC12970	3FLAG_ygaC_seq_F	GTCGCAAATATGATGGTTATACC
OLEC12971	3FLAG_ygaC_seq_R	CTTGGAAAAGTTTATGCAGAC
**Deletion of *yebC* and *speB* in *S. pyogenes***		
OLEC13193	ko_yebC_up_EcoRI_F	ACACAGAATTCAAACGCTATACAAAAGCTCG
OLEC13194	ko_yebC_up_KpnI_R	ACACAGGTACCTTTTGTCTCCTTTTAATAGTATTTTATTG
OLEC12920	ko_yebC_dw_BamHI_F	ACACAGGATCCATTGACATAGAATAATAAAAAGAGTTG
OLEC13195	ko_yebC_dw_SalI_R	ACACAGTCGACACTCAAATTGGTTAACATTTGAG
OLEC13196	ko_yebC_seq_F	AATGATGAATTGGCAAGTCG
OLEC12923	3FLAG_ko_yebC_seq_R	TCCATCAGCAGTCAACTC
OLEC15289	ko_speB_up_EcoRI_F	ACACAGAATTCCATAAGGTCAATAGCCAGATGC
OLEC15290	ko_speB _up_KpnI_R	ACACAGGTACCTCAAAATAAGTTAATCTACTGCATTTGC
OLEC15291	ko_speB _dw_BamHI_F	ACACAGGATCCATGGAAATGCATTTCGTTAGAAC
OLEC15292	ko_speB _dw_PaeI_R	ACACAGCATGCGAGCCTCTTTTCTCATTAATAATTACTG
OLEC15293	ko_speB _seq_F	TTGATGTCAAAAATACGTTACGC
OLEC15294	ko_speB _seq_R	GCTGATAAGGTCACGACTGTC
**Complementation of *yebC* in *S. pyogenes***		
OLEC13350	comp_p7INT_BamHI_F	ACACAGGATCCCATTAATGAATCGGCCAAC
OLEC13351	comp_p7INT_XbaI_R	ACACATCTAGACGTAATAGCGAAGAGGC
OLEC13346	comp_yebC_BamHI_F	ACACAGGATCCGCTGTTGTCACTCAAGC
OLEC13347	comp_yebC_XbaI_R	ACACATCTAGACAACGACTATGTTACCTGG
OLEC13348	comp_yebC_3FLAG_F	TCATGATATCGACTACAAAGATGACGACGATAAATAGTAAATTGACATAGA ATAATAAAAAG
OLEC13349	comp_yebC_3FLAG_R	TCTTTATAATCACCGTCATGGTCTTTGTAGTCGGTACCAAAATCTGCTACA TTATGATA
**Deletion of *efp, yeeN and yebC* and depletion of *yebC* using CRISPRi in *S.* Typhimurium**		
3864	5'-Defp-tetR_fw	CCAGTTAACAATTTCAGAGGGCCTTATGGCGACTTACTATTTAAGACCCAC TTTCACATT
3865	3'-Defp-tetA_rv	GCACCATTTTTCCCGATAACGTAAATTATTTTACGCGGGACTAAGCACTTG TCTCCTG
3866	5'-efp_seq_56C_fw	AAGAGGATCGTCAGGGTTGC
3867	3'-Defp clean_51C_rv	GCACCATTTTTCCCGATAACGTAAATTATTTTACGCGGGAATAGTAAGTCG CCATAAGGC
8341	5’-DyeeN-KanSceI_fw	TTAATGTAAATAATTTTTTGAGGAGATGTTTCCAGTGGGAAGGGTTTTCCC AGTCACGAC
8342	3’-DyeeN-KanSceI_rv	AGAGACAGATTTGTTTAATTTATAATTAGAGGTTTGCAACTGCTTCCGGCT CGTATGTTG
8343	5’-DyeeN-clean_fw	TAACCTGCCATTAATGTAAATAATTTTTTGAGGAGATGTTTCCAGTGGGAG TTGCAAACC
8344	3’-DyeeN-clean_rv	AAATCTGCCATAGAGACAGATTTGTTTAATTTATAATTAGAGGTTTGCAACT CCCACTGG
8345	5’-yeeN-seq_fw	CCACAATATGTCGCAAGACG
8346	3’-yeeN-seq_rv	GCTATGTGTCAGATCAGGTC
8347	5’-DyebC-KanSceI_fw	GCGCGCTTTTTTTGAGGAAATATTTATGGCAGGTCATAGTAGGGTTTTCCC AGTCACGAC

8348	3’-DyebC-KanSceI-rv	CATCGTCACAGTCTTCCAGCATATCGATCAGACGTAGCAGTGCTTCCGGC TCGTATGTTG
8349	5’-DyebC-clean_fw	GCCTGAAAAGGCGCGCTTTTTTTGAGGAAATATTTATGGCAGGTCATAGT CTGCTACGTC
8350	3’-DyebC-clean_rv	CTTCCTGGACATCGTCACAGTCTTCCAGCATATCGATCAGACGTAGCAGA CTATGACCTG
8351	5’-yebC-seq_fw	CACTGAACATCTGACGTACC
8352	3’-yebC-seq_rv	TAGGTCAGTTGTCTGCCTAC
OLEC15303	gRNA-yebC-nts_F	/5phos/AAACACACCAGACATCGTAAAGCT
OLEC15304	gRNA-yebC-nts_R	/5phos/CCATAGCTTTACGATGTCTGGTGT
**Site-directed mutagenesis of *yebC* in *S. pyogenes***		
OLEC14266	SDM_yebC_m1_F	TGCCGCATTTGGTGTTGAAATTTATGTG
OLEC14267	SDM_yebC_m1_R	TAGACAGCTGATGTTGCTCCATCTTTAG
OLEC14268	SDM_yebC_m3_F	GCTGCGATTGATGCAGCTAAAGGAAACACAGATGAAAC
OLEC14269	SDM_yebC_m3_R	ATCAATAACTGCAGCTGGCACTTGTGCTTGCTT
OLEC14270	SDM_yebC_m4_F	GCAACGGCTTACGGTGCTAACGGTGGCAATATGGGA
OLEC14271	SDM_yebC_m4_R	TACATTTGCCGCTGTAGCGTTAACATTTGATGTCAAAGTATCC
OLEC14388	SDM_yebC_m5_F	TGCACAATTACTTGCTGCGGATGTAGACGTAGATG
OLEC14389	SDM_yebC_m5_R	AAGACAGCTGCAGCAGCATCACCAGCAAAAACGATG
OLEC14392	SDM_yebC_m6_F	TGCAACTTTTGCAAAGCTTATTGATGCACTTG
OLEC14393	SDM_yebC_m6_R	AGGTCAGCACCTGCCAAAACTACTTCTGATTGAG
OLEC14272	SDM_yebC_Y84A_F	AGAGGGACGCGCTGAAGGTTTTG
OLEC14273	SDM_yebC_Y84A_Y84F_R	ACGAAAGTTTCATCTGTG
OLEC14387	SDM_yebC_Y84F_F	AGAGGGACGCTTTGAAGGTTTTG
OLEC14274	SDM_yebC_E85A_F	GGGACGCTATGCTGGTTTTGGTC
OLEC14275	SDM_yebC_E85A_R	TCTACGAAAGTTTCATCTG
speB* reporters in *S. pyogenes		
OLEC15391	HiFi_pSpeB_F	GCCGATTCATTAATGGGATCCTGTGTTTGATGTCAAAAATACGTTACGC
OLEC15287	HiFi_pSpeB_R	TGAAAAGTTCTTCTCCTTTGCTCATTTTTTTTATACCTCTTTCAAAATAAGTT AATCTAC
OLEC15288	HiFi_GFP_F	ATGAGCAAAGGAGAAGAACTTTTCAC
OLEC13350	HiFi_GFP_R	ACACAGGATCCCATTAATGAATCGGCCAAC
OLEC15328	HiFi_speB_F	CTAGTTAAGGAGGTGATCTCATATGAATAAAAAGAAATTAGGTGTCAG
OLEC15329	HiFi_speB_R	GTTTTTCTGTCGCTTTTAGCCATTAAGGTTTGATGCCTACAACAG
OLEC15325	HiFi_pTet_F	TAATGGCTAAAAGCGACAGAAAAAC
OLEC14631	HiFi_pTet_R	CATATGAGATCACCTCCTTAACTAG
**iCLIP2 for YebC in *S. pyogenes***		
OLEC14114	RToligo	GGATCCTGAACCGCT
OLEC14115	L3-App	/rApp/AGATCGGAAGAGCGGTTCAG/ddC/
OLEC14167	L07clip2.0	/5Phos/NNNNCAGATCNNNNNAGATCGGAAGAGCGTCGTG/3ddC/
OLEC14168	L08clip2.0	/5Phos/NNNNACTTGANNNNNAGATCGGAAGAGCGTCGTG/3ddC/
OLEC14169	L09clip2.0	/5Phos/NNNNGATCAGNNNNNAGATCGGAAGAGCGTCGTG/3ddC/
OLEC14170	L10clip2.0	/5Phos/NNNNTAGCTTNNNNNAGATCGGAAGAGCGTCGTG/3ddC/

OLEC14171	L11clip2.0	/5Phos/NNNNATGAGCNNNNNAGATCGGAAGAGCGTCGTG/3ddC/
OLEC14172	L12clip2.0	/5Phos/NNNNCTTGTANNNNNAGATCGGAAGAGCGTCGTG/3ddC/
OLEC14120	P5Solexa_s	ACACGACGCTCTTCCGATCT
OLEC14121	P3Solexa_s	CTGAACCGCTCTTCCGATCT
OLEC14122	P5Solexa	AATGATACGGCGACCACCGAGATCTACACTCTTTCCCTACACGACGCTCT TCCGATCT
OLEC14123	P3Solexa	CAAGCAGAAGACGGCATACGAGATCGGTCTCGGCATTCCTGCTGAACCG CTCTTCCGATCT
OLEC14202	Seq_1	ACACTCTTTCCCTACACGACGCTCTTCCGATCT
OLEC14203	Seq_2	CGGTCTCGGCATTCCTGCTGAACCGCTCTTCCGATCT
**Ribosome profiling**		
OLEC12113	ribseq_15_control (RNA)	AUGUACACGGAGUCG
OLEC12114	ribseq_45_control (RNA)	AUGUACACGGAGUCGACCCGCAACGCGAUGUACACGGAGUCGACC
OLEC12115	ribseq_adapter_with_UMI	/rApp/NNNNNATCGTAGATCGGAAGAGCACACGTCTGAA/3ddC/
OLEC12116	ribseq_RT_primer	/5Phos/NNAGATCGGAAGAGCGTCGTGTAGGGAAAGAG/iSp18/GTGACTG GAGTTCAGACGTGTGCTC
OLEC12244	ribseq_libamp_fw	AATGATACGGCGACCACCGAGATCTACACTCTTTCCCTACACGACGCTC
OLEC12245	ribseq_libamp_rev_ind_1	CAAGCAGAAGACGGCATACGAGATCGTGATGTGACTGGAGTTCAGACGT GTG
OLEC12246	ribseq_libamp_rev_ind_2	CAAGCAGAAGACGGCATACGAGATACATCGGTGACTGGAGTTCAGACGT GTG
OLEC12247	ribseq_libamp_rev_ind_3	CAAGCAGAAGACGGCATACGAGATGCCTAAGTGACTGGAGTTCAGACGT GTG
OLEC14922	ribseq_libamp_rev_ind_4	CAAGCAGAAGACGGCATACGAGATTGGTCAGTGACTGGAGTTCAGACGT GTG
OLEC14923	ribseq_libamp_rev_ind_5	CAAGCAGAAGACGGCATACGAGATCACTGTGTGACTGGAGTTCAGACGT GTG
OLEC14924	ribseq_libamp_rev_ind_6	CAAGCAGAAGACGGCATACGAGATATTGGCGTGACTGGAGTTCAGACGT GTG
OLEC14925	ribseq_libamp_rev_ind_7	CAAGCAGAAGACGGCATACGAGATGATCTGGTGACTGGAGTTCAGACGT GTG
OLEC14926	ribseq_libamp_rev_ind_8	CAAGCAGAAGACGGCATACGAGATTCAAGTGTGACTGGAGTTCAGACGT GTG
OLEC14927	ribseq_libamp_rev_ind_9	CAAGCAGAAGACGGCATACGAGATCTGATCGTGACTGGAGTTCAGACGT GTG
OLEC14928	ribseq_libamp_rev_ind_10	CAAGCAGAAGACGGCATACGAGATAAGCTAGTGACTGGAGTTCAGACGT GTG
OLEC14929	ribseq_libamp_rev_ind_11	CAAGCAGAAGACGGCATACGAGATGTAGCCGTGACTGGAGTTCAGACGT GTG
OLEC14930	ribseq_libamp_rev_ind_12	CAAGCAGAAGACGGCATACGAGATTACAAGGTGACTGGAGTTCAGACGT GTG
**Ribosome stalling reporter in *S. pyogenes***		
OLEC15067	P5_P3_R	TGGAGGTGGTCCTTTGTAGAGCTCATCCATG
OLEC15068	P5_F	CCTCCATCTGTTTCAGAACTTATCAAAGAAAAC
OLEC15120	link_F	TCTGTTTCAGAACTTATCAAAGAAAACATGC
OLEC15122	stop_R	TTATCCTTTGTAGAGCTCATCCATGCCATGTG
OLEC15140	PPG_R	TCCAGGTGGTCCTTTGTAGAGCTC
OLEC15141	PIP_R	TGGAATTGGTCCTTTGTAGAGCTCATCC
**Ribosome stalling reporter *in vitro***		
OLEC14889	HiFi_p21_F	CATATGTATATCTCCTTCTTAAAGTTAAAC
OLEC14890	HiFi_p21_R	CTCGAGCACCACCACCAC
OLEC14953	HiFi_b1983_F	TTAAGAAGGAGATATACATATGGGACGTAAATGGGCC
OLEC14954	HiFi_b1983_R	AGTGGTGGTGGTGGTGGTGCTCGAGGAGATTTGCGACGTTATGATAAAC

OLEC15057	HiFi_b1864_F	TTAAGAAGGAGATATACATATGGCAGGTCATAGTAAATGGGC
OLEC15058	HiFi_b1864_R	AGTGGTGGTGGTGGTGGTGCTCGAGGAGAGTCGCTGCGACC
OLEC15015	b1983_HA_F	ATCGTATGGGTAGGTACCGAGATTTGCGACGTTATGATAAAC
OLEC15016	b1983_b1864_HA_R	GTTCCAGATTACGCTCATCACCACCACCACCACTGAG
OLEC15093	b1864_HA_F	ATCGTATGGGTAGGTACCGAGAGTCGCTGCGACCTC
OLEC15063	b1983_m2_1_F	GCTGCAATTGATGCAGCCAAAGGCGGCG
OLEC15064	b1983_m2_1_R	ATCAATAACAGCTGCTGGAACTTGTGCCTGC
OLEC15065	b1983_m2_2_F	ACAATTTTCAATGCAAAAGGCGGCAATATCGG
OLEC15066	b1983_m2_2_R	TGCAACGTTAGCAATCGTAGCGTTAACATTTGAAGTC
OLEC15073	b1983_Y84A_F	GCAGAAGGCTTTGGTCCTAATGGC
OLEC15074	b1983_Y84A_R	ACGTCCCTGCACGAAC
OLEC15051	folA_FLAG_F	ATCAGTCTGATTGCGGC
OLEC14962	folA_FLAG_R	CTTGTCGTCATCGTCTTTGTAGTCCATATGTATATCTCCTTCTTAAAGTTAAAC
OLEC15076	folA_P0_F	GCGGGACGCAAAAATATTATCCTCAGC
OLEC15165	folA_P0_R	CAAGGCACGACCGATTGATTCCCAG
OLEC15054	folA_P5_F	TGGTGGCGGACGACCGATTGATTC
OLEC15055	folA_P5_R	CCGCCAGGACGCAAAAATATTATCCTCAG
OLEC14961	folA_stop_F	TAATGAGGATCCCGGGAATTC
OLEC15052	folA_stop_R	CAACGGACGACCGATTGATTC
**qRT-PCR**		
OLEC15305	q-yebC_F	GCGTCATGCTTTCAGCAAGTG
OLEC15306	q-yebC_R	GCACTTTGCCCATCTCTTCC
OLEC15307	q-16S_F	CTTCGGGTTGTAAAGTACTTTCAGC
OLEC15308	q-16S_R	TGCAGTTCCCAGGTTGAGC
718	q-gmk_F	TTTTGCCGCCGTCAAAGATC
719	q-gmk_R	ATGGCTCATTTCTGCAACCG

Supplementary table 2: Plasmids used in the study

Code	Description
**Introduction of 3x FLAG tag to the C-termini of *S. pyogenes* genes**	
pEC2818	pSEVA141-3xFLAG-lox71-erm-lox66
pEC2819	pSEVA141-yhaM-3xFLAG-lox71-erm-lox66
pEC2820	pSEVA141-gapN-3xFLAG-lox71-erm-lox66
pEC2969	pSEVA141-yebC-3xFLAG-lox71-erm-lox66
pEC2972	pSEVA141-yjbK-3xFLAG-lox71-erm-lox66
pEC2974	pSEVA141-ygaC-3xFLAG-lox71-erm-lox66
pEC3034	pSEVA141-phoH-3xFLAG-lox71-erm-lox66
pEC3035	pSEVA141-thuC-3xFLAG-lox71-erm-lox66
pEC455	repDEG-pAMbeta1-pJH1-aphIII-bgaB-colE1-PgyrA-Cre

yebC* mutants in *S. pyogenes	
pEC2817	pSEVA141-lox71-erm-lox66
pEC2976	pSEVA141-yebC_lox71-erm-lox66
pEC2988	p7INTΔlacZα_Spy0316-3xFLAG
pEC3071	p7INTΔlacZα_Spy0316(K21A, K25A)-3xFLAG
pEC3085	p7INTΔlacZα_Spy0316(K61A, H62A, K66A, K70A, R105A, R111A, K116A)-3xFLAG
pEC3072	p7INTΔlacZα_Spy0316(K61A, H62A, K66A, K70A)-3xFLAG
pEC3073	p7INTΔlacZα_Spy0316(R105A, R111A, K116A)-3xFLAG
pEC3087	p7INTΔlacZα_Spy0316(D143A, D145A, S146A, E149A, E153A)-3xFLAG
pEC3089	p7INTΔlacZα_Spy0316(E208A, D210A, E213A, E216A)-3xFLAG
pEC3074	p7INTΔlacZα_Spy0316(Y84A)-3xFLAG
pEC3086	p7INTΔlacZα_Spy0316(Y84F)-3xFLAG
pEC3075	p7INTΔlacZα_Spy0316(E85A)-3xFLAG
**Deletion of *efp, yeeN and yebC* and depletion of *yebC* using CRISPRi in *S.* Typhimurium**	
-	pWRG730
-	pdCas9-sgRNA-RFP
-	pdCas9-sgRNA-yebC
speB* reporters in *S. pyogenes	
pEC3180	pSEVA141-speB_lox71-erm-lox66
pEC3183	p7INTΔlacZα_pTet_sfGFP
pEC3181	p7INTΔlacZα_pTet_speB
pEC3182	p7INTΔlacZα_pSpeB_sfGFP
**Ribosome stalling reporter in *S. pyogenes***	
pEC3155	p7INTΔlacZα_pTet_3xFLAG-sfGFP-P5-mKate
pEC3156	p7INTΔlacZα_pTet_3xFLAG-sfGFP-P3-mKate
pEC3157	p7INTΔlacZα_pTet_3xFLAG-sfGFP-PPG-mKate
pEC3158	p7INTΔlacZα_pTet_3xFLAG-sfGFP-PIP-mKate
pEC3160	p7INTΔlacZα_pTet_3xFLAG-sfGFP-P0-mKate
pEC3161	p7INTΔlacZα_pTet_3xFLAG-sfGFP-stop-mKate
**Ribosome stalling reporter *in vitro***	
pEC3162	pET21-a(+)_b1983:HA tag
pEC3163	pET21-a(+)_b1983(K61A, H62A, K66A, K70A, R105A, R111A, K116A):HA tag
pEC3164	pET21-a(+)_b1983(Y84A):HA tag
pEC3165	pET21-a(+)_b1864:HA tag

Supplementary table 3: Strains of *S. pyogenes* used in the study

Code	Strain	Genotype
**The wild type strain**		
EC2224	*WT*	SF370 (M1 serotype)
**Strains with the 3x FLAG tag-labelled candidate RBPs**		
EC3638	*yhaM::3xFLAG*	SPy_0267::3xFLAG-lox72
EC3639	*gapN::3xFLAG*	SPy_1371::3xFLAG-lox72
EC3640	*yebC::3xFLAG*	SPy_0316::3xFLAG-lox72
EC3646	*phoH::3xFLAG*	SPy_0471::3xFLAG-lox72
EC3647	*thuC::3xFLAG*	SPy_0539::3xFLAG-lox72
EC3707	*yjbK::3xFLAG*	SPy_1124::3xFLAG-lox72
EC3641	*ygaC::3xFLAG*	SPy_1608::3xFLAG-lox72
**Strains with *yebC* deletion and complementation**		
EC3615	Δ*yebC*	ΔSPy_0316::lox72
EC3619	Δ*yebC / yebC+*	ΔSPy_0316::lox72 SPy_S01::p7INTΔlacZα_SPy_0316::3xFLAG
EC3690	Δ*yebC / yebC_M1*	ΔSPy_0316::lox72 SPy_S01::p7INTΔlacZα_SPy_0316(K21A, K25A):3xFLAG
EC3691	Δ*yebC / yebC_M3*	ΔSPy_0316::lox72 SPy_S01::p7INTΔlacZα_SPy_0316(K61A, H62A, K66A, K70A):3xFLAG
EC3692	Δ*yebC / yebC_M4*	ΔSPy_0316::lox72 SPy_S01::p7INTΔlacZα_SPy_0316(R105A, R111A, K116A):3xFLAG
EC3693	Δ*yebC / yebC_Y84A*	ΔSPy_0316::lox72 SPy_S01::p7INTΔlacZα_SPy_0316(Y84A):3xFLAG
EC3694	Δ*yebC / yebC_E85A*	ΔSPy_0316::lox72 SPy_S01::p7INTΔlacZα_SPy_0316(E85A):3xFLAG
EC3714	Δ*yebC / yebC_M2*	ΔSPy_0316::lox72 SPy_S01::p7INTΔlacZα_SPy_0316(K61A, H62A, K66A, K70A, R105A, R111A, K116A):3xFLAG
EC3715	Δ*yebc / yebC_Y84F*	ΔSPy_0316::lox72 SPy_S01::p7INTΔlacZα_SPy_0316(Y84F):3xFLAG
EC3716	Δ*yebC / yebC_M5*	ΔSPy_0316::lox72 SPy_S01::p7INTΔlacZα_SPy_0316(D143A, D145A, S146A, E149A, E153A):3xFLAG
EC3718	Δ*yebC / yebC_M6*	ΔSPy_0316::lox72 SPy_S01::p7INTΔlacZα_SPy_0316(E208A, D210A, E213A, E216A):3xFLAG
**Reporter strains for *speB* regulation**		
EC3837	Δ*speB*	ΔSPy_2039::lox72
EC3838	Δ*yebC* Δ*speB*	ΔSPy_0316::lox72 ΔSPy_2039::lox72
EC3839	Δ*speB pTet-speB*	ΔSPy_2039::lox72 Spy_s01::p7INTΔlacZα_Ptet_SPy_2039
EC3840	Δ*yebC* Δ*speB pTet-speB*	ΔSPy_0316::lox72 ΔSPy_2039::lox72 Spy_s01::p7INTΔlacZα_Ptet_Spy_2039
EC3841	*WT pSpeB-sfGFP*	Spy_s01::p7INTΔlacZα_Pspy_2039_sfGFP
EC3842	Δ*yebC pSpeB-sfGFP*	ΔSPy_0316::lox72 Spy_s01::p7INTΔlacZα_Pspy_2039_sfGFP
EC3843	*WT pTet-sgGFP*	Spy_s01::p7INTΔlacZα_Ptet_sfGFP
EC3844	Δ*yebC pTet-sgGFP*	ΔSPy_0316::lox72 Spy_s01::p7INTΔlacZα_Ptet_sfGFP
**Reporter strains for the ribosome stalling**		
EC3788	*WT sfGFP-P5-mKate*	Spy_s01::p7INTΔlacZα_pTet_3xFLAG-sfGFP-P5-mKate
EC3789	*WT sfGFP-P3-mKate*	Spy_s01::p7INTΔlacZα_pTet_3xFLAG-sfGFP-P3-mKate
EC3790	*WT sfGFP-PPG-mKate*	Spy_s01::p7INTΔlacZα_pTet_3xFLAG-sfGFP-PPG-mKate
EC3791	*WT sfGFP-PIP-mKate*	Spy_s01::p7INTΔlacZα_pTet_3xFLAG-sfGFP-PIP-mKate
EC3794	Δ*yebC sfGFP-P5-mKate*	ΔSPy_0316 Spy_s01::p7INTΔlacZα_pTet_3xFLAG-sfGFP-P5-mKate

EC3795	Δ*yebC sfGFP-P3-mKate*	ΔSPy_0316 Spy_s01::p7INTΔlacZα_pTet_3xFLAG-sfGFP-P3-mKate
EC3796	Δ*yebC sfGFP-PPG-mKate*	ΔSPy_0316 Spy_s01::p7INTΔlacZα_pTet_3xFLAG-sfGFP-PPG-mKate
EC3797	Δ*yebC sfGFP-PIP-mKate*	ΔSPy_0316 Spy_s01::p7INTΔlacZα_pTet_3xFLAG-sfGFP-PIP-mKate
EC3799	Δ*yebC sfGFP-P0-mKate*	ΔSPy_0316 Spy_s01::p7INTΔlacZα_pTet_3xFLAG-sfGFP-P0-mKate
EC3800	Δ*yebC sfGFP-stop-mKate*	ΔSPy_0316 Spy_s01::p7INTΔlacZα_pTet_3xFLAG-sfGFP-stop-mKate

Supplementary table 4: Strains of *S.* Typhimurium used in the study

Code	Strain	Genotype
**The wild type strain**		
TH437	WT	LT2 (from J. Roth stab)
***S.* Typhimurium strains with *efp*, *yeeN* and *yebC* deletion**		
EM9876	Δ*efp*	Δ*efp* (leaving first and last 15 bp)
EM17605	Δ*yeeN*	Δ*yeeN* (Δaa5-aa237)
EM17606	Δ*yebC*	Δ*yebC* (Δaa5-aa215)
EM17607	Δ*efp*	Δ*efp* (leaving first and last 15 bp) Δ*yeeN* (Δaa5-aa237)
EM17608	Δ*yeeN* Δ*yebC*	Δ*yeeN* (Δaa5-aa237) Δ*yebC* (Δaa5-aa215)
***S*. Typhimurium strains with CRISPRi plasmids**		
EM18648	WT / *yebC*	LT2 / pdCas9-sgRNA-yebC_nts
EM18649	WT / VC	LT2 / pdCas9-sgRNA-RFP
EM18650	Δ*efp* / *yebC*	Δ*efp* (leaving first and last 15 bp) / pdCas9-sgRNA-yebC_nts
EM18651	Δ*efp* / VC	Δ*efp* (leaving first and last 15 bp) / pdCas9-sgRNA-RFP
EM18652	Δ*yebC* / *yebC*	Δ*yebC* (Δaa5-aa215) / pdCas9-sgRNA-yebC_nts
EM18653	Δ*yebC* / VC	Δ*yebC* (Δaa5-aa215) / pdCas9-sgRNA-RFP
EM18654	Δ*efp* Δ*yeeN* / *yebC*	Δ*efp* (leaving first and last 15 bp) Δ*yeeN* (Δaa5-aa237) / pdCas9-sgRNA-yebC_nts
EM18655	Δ*efp* Δ*yeeN* / VC	Δ*efp* (leaving first and last 15 bp) Δ*yeeN* (Δaa5-aa237) / pdCas9-sgRNA-RFP
