# Supplementary material for: RNA-binding protein YebC enhances translation of proline-rich amino acid stretches in bacteria
Source: Nat Commun. 2025 Jul 7;16:6262. doi: 10.1038/s41467-025-60687-4 (PMC12234827; doi:10.1038/s41467-025-60687-4)
Supplement: Supplementary file 2 — Description of Additional Supplementary Files [file 41467_2025_60687_MOESM2_ESM.pdf]

Description of Additional Supplementary Files:

Supplementary Data 1: Results of OOPS and RBS-ID experiments.

Supplementary Data 2: Annotated RBPs in *S. pyogenes*.

Supplementary Data 3: RNA-seq of WT, Δ*yebC* and Δ*yebC* / *yebC*+ strains in mid-logarithmic and stationary growth phases.

Supplementary Data 4: Clusters of cross-linked nucleotides in iCLIP experiment.

Supplementary Data 5: Ribosome profiling: pause scores for codons with changed pausing in the *yebC* mutant strains.

Supplementary Data 6: Ribosome profiling: genomic loci with increased pausing in the *yebC* mutants.

Supplementary Data 7: MS proteomics of WT, Δ*yebC* and Δ*yebC* / *yebC*+ strains in mid-logarithmic and stationary growth phases.
