# Supplementary material for: RNA-binding protein YebC enhances translation of proline-rich amino acid stretches in bacteria
Source: Nat Commun. 2025 Jul 7;16:6262. doi: 10.1038/s41467-025-60687-4 (PMC12234827; doi:10.1038/s41467-025-60687-4)
Supplement: Supplementary file 10 — Reporting Summary [file 41467_2025_60687_MOESM10_ESM.pdf]

nature portfolio

Corresponding author(s): Emmanuelle Charpentier

Last updated by author(s): Mar 22, 2025

# Reporting Summary

Nature Portfolio wishes to improve the reproducibility of the work that we publish. This form provides structure for consistency and transparency in reporting. For further information on Nature Portfolio policies, see our Editorial Policies and the Editorial Policy Checklist.

## Statistics

For all statistical analyses, confirm that the following items are present in the figure legend, table legend, main text, or Methods section.

| n/a | Confirmed | |
|---|---|---|
| ☐ | ☒ | The exact sample size (*n*) for each experimental group/condition, given as a discrete number and unit of measurement |
| ☐ | ☒ | A statement on whether measurements were taken from distinct samples or whether the same sample was measured repeatedly |
| ☐ | ☒ | The statistical test(s) used AND whether they are one- or two-sided<br>*Only common tests should be described solely by name; describe more complex techniques in the Methods section.* |
| ☒ | ☐ | A description of all covariates tested |
| ☐ | ☒ | A description of any assumptions or corrections, such as tests of normality and adjustment for multiple comparisons |
| ☐ | ☒ | A full description of the statistical parameters including central tendency (e.g. means) or other basic estimates (e.g. regression coefficient) AND variation (e.g. standard deviation) or associated estimates of uncertainty (e.g. confidence intervals) |
| ☐ | ☒ | For null hypothesis testing, the test statistic (e.g. *F*, *t*, *r*) with confidence intervals, effect sizes, degrees of freedom and *P* value noted<br>*Give P values as exact values whenever suitable.* |
| ☒ | ☐ | For Bayesian analysis, information on the choice of priors and Markov chain Monte Carlo settings |
| ☒ | ☐ | For hierarchical and complex designs, identification of the appropriate level for tests and full reporting of outcomes |
| ☐ | ☒ | Estimates of effect sizes (e.g. Cohen's *d*, Pearson's *r*), indicating how they were calculated |

*Our web collection on statistics for biologists contains articles on many of the points above.*

## Software and code

Policy information about availability of computer code

| | |
|---|---|
| Data collection | OD measurement of bacterial cultures: Gen5 v. 2.09<br>Phosphor imaging: Typhoon FLA9500 version 1.1<br>Western blotting: Fusion Software v18-02<br>qRT-PCR: QuantStudio Design & Analysis Software v. 1.5.3<br>Microscopy: Leica Application Suite X 3.7.6.25997<br>Sucrose gradients: BioComp FlowCell Software<br>Mass spectrometry: Orbitrap Exploris 480 Tune Application: 4.2.362.42<br>Mass spectrometry: Orbitrap Fusion Lumos Tune Application: 4.1.4244<br>RNA-seq: NovaSeq Control Software v1.8 |
| Data analysis | GraphPad Prism version 9.0.0<br>ImageJ 2.1.0<br>MicrobeJ version 5.10n<br>MSFragger (version 3.0) within FragPipe (version 13.0)<br>Spectronaut® (Biognosys AG, Zurich, Switzerland) Version 17<br>MSFragger (version 3.0)<br>eggNOG-mapper (v2.1.12)<br>PyMOL Molecular Graphics System, Version 3.0<br>NovaSeq Control Software v1.8 |

Cutadapt v4.4
STAR RNA-seq aligner v2.7.3a
Samtools v1.19.2
Bedtools v2.31.0
UMI Tools v1.1.5
featureCounts v2.0.1
DESeq2 v1.38.0
Snakemake v8.4.8
FASTX-Toolkit v0.0.13
seqtk subseq v1.3
UCSF ChimeraX 1.8
Flexbar v3.5.0
R programming language version 4.3.1
MSstats v4.14.2
stringr v1.5.0
tidyverse v2.0.0
VennDiagram v1.7.3
beeswarm v0.4.0
ggpubr v0.6.0
ggrepel v0.9.4
plotROC v2.3.1
UniProt.ws v2.40.1
janitor v2.2.1
drawProteins v1.20.0
ggvenn v0.1.10
GenomicAlignments v1.36.0
edgeR v3.42.4
heatmaply v1.5.0
Biostrings v2.68.1
ggseqlogo v0.2.0

Source code:
- https://github.com/MPUSP/ignatov_et_al_2025 [https://doi.org/10.5281/zenodo.15401960]
- https://github.com/MPUSP/snakemake-bacterial-riboseq [https://doi.org/10.5281/zenodo.15403357]

For manuscripts utilizing custom algorithms or software that are central to the research but not yet described in published literature, software must be made available to editors and reviewers. We strongly encourage code deposition in a community repository (e.g. GitHub). See the Nature Portfolio guidelines for submitting code & software for further information.

## Data

Policy information about availability of data

All manuscripts must include a data availability statement. This statement should provide the following information, where applicable:

- Accession codes, unique identifiers, or web links for publicly available datasets
- A description of any restrictions on data availability
- For clinical datasets or third party data, please ensure that the statement adheres to our policy

All next generation sequencing data have been deposited in the European Nucleotide Archive (ENA) under the accession PRJEB78417. Processed alignment files of the ribosome profiling footprints and the iCLIP data have been deposited in the Open Research Repository (EDMOND) of the Max Planck Society [https://doi.org/10.17617/3.8PZNYF]. The proteomics mass spectrometry data have been deposited to the ProteomeXchange Consortium via the PRIDE partner repository with the dataset identifier PXD054642. The source code to reproduce the main results reported in this study are available at https://github.com/MPUSP/ignatov_et_al_2025 [https://doi.org/10.5281/zenodo.15401960]. The source code for the ribo-seq pipeline is available at https://github.com/MPUSP/snakemake-bacterial-riboseq [https://doi.org/10.5281/zenodo.15403357]. Source Data are provided with this paper.

## Research involving human participants, their data, or biological material

Policy information about studies with human participants or human data. See also policy information about sex, gender (identity/presentation), and sexual orientation and race, ethnicity and racism.

| | |
|---|---|
| Reporting on sex and gender | n/a |
| Reporting on race, ethnicity, or other socially relevant groupings | n/a |
| Population characteristics | n/a |
| Recruitment | n/a |
| Ethics oversight | n/a |

Note that full information on the approval of the study protocol must also be provided in the manuscript.

# Field-specific reporting

Please select the one below that is the best fit for your research. If you are not sure, read the appropriate sections before making your selection.

- [x] Life sciences
- [ ] Behavioural & social sciences
- [ ] Ecological, evolutionary & environmental sciences

For a reference copy of the document with all sections, see nature.com/documents/nr-reporting-summary-flat.pdf

# Life sciences study design

All studies must disclose on these points even when the disclosure is negative.

| | |
|---|---|
| Sample size | No sample size calculations were performed. The number of replicates for each experiment was sufficient for hypothesis testing. |
| Data exclusions | No data were excluded from the analyses |
| Replication | Most experiments were performed in three or more biological replicates. Some qualitative experiments were performed in two biological replicates and their results were reproducible. The iCLIP control libraries "WT_UV+" and "YebC_UV-" were prepared and sequenced one time. |
| Randomization | Randomization and control of covariates were not required in our study. |
| Blinding | The results of experiments were analyzed using methods not sensitive to human judgment and therefore blinding was not relevant. |

# Reporting for specific materials, systems and methods

We require information from authors about some types of materials, experimental systems and methods used in many studies. Here, indicate whether each material, system or method listed is relevant to your study. If you are not sure if a list item applies to your research, read the appropriate section before selecting a response.

## Materials & experimental systems

| n/a | Involved in the study | |
|---|---|---|
| [ ] | [x] | Antibodies |
| [x] | [ ] | Eukaryotic cell lines |
| [x] | [ ] | Palaeontology and archaeology |
| [x] | [ ] | Animals and other organisms |
| [x] | [ ] | Clinical data |
| [x] | [ ] | Dual use research of concern |
| [x] | [ ] | Plants |

## Methods

| n/a | Involved in the study | |
|---|---|---|
| [x] | [ ] | ChIP-seq |
| [x] | [ ] | Flow cytometry |
| [x] | [ ] | MRI-based neuroimaging |

## Antibodies

| | |
|---|---|
| Antibodies used | Streptococcus pyogenes speB Polyclonal Antibody (Thermo Scientific; PA5-117551; Lot # ZE4322779)<br>Monoclonal ANTI-FLAG M2 antibody produced in mouse (Merck; F1804; Source # SLCK5688)<br>HA Tag Monoclonal Antibody (Thermo Scientific; #26183; Lot # YF373680) |
| Validation | In western blotting, the MW of the detected proteins corresponds to the expected. The signals are not visible in the negative controls. |

## Plants

| | |
|---|---|
| Seed stocks | n/a |
| Novel plant genotypes | n/a |
| Authentication | n/a |
