# Supplementary material for: RNA-binding protein YebC enhances translation of proline-rich amino acid stretches in bacteria
Source: Nat Commun. 2025 Jul 7;16:6262. doi: 10.1038/s41467-025-60687-4 (PMC12234827; doi:10.1038/s41467-025-60687-4)

Supplementary figure 9: Association of YebC with ribosomes

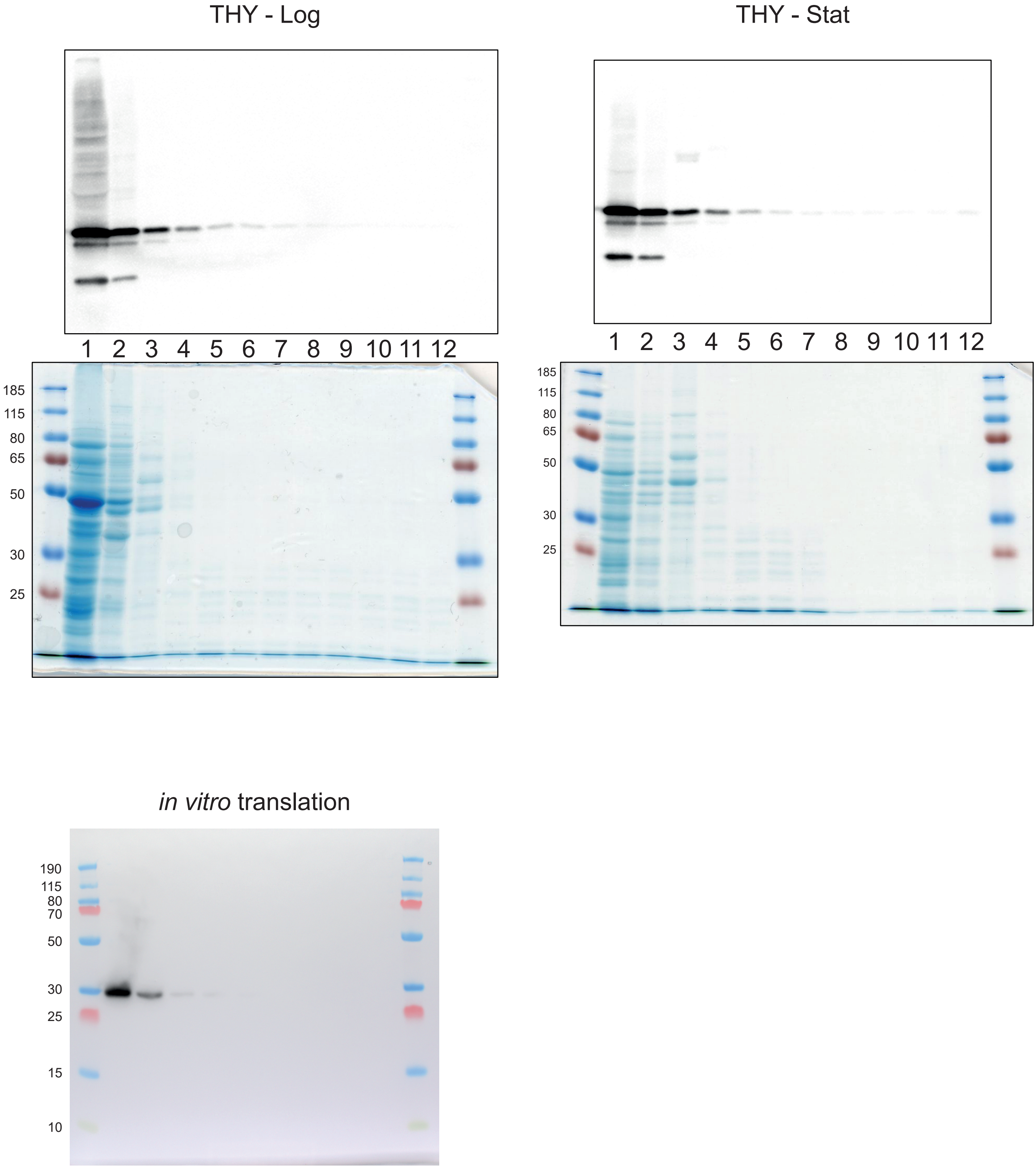

Supplement: Supplementary file 12 — Source Data [file 41467_2025_60687_MOESM12_ESM.zip › 250519_Source_Data/Fig_S9_WB_YebC_ribosome.pdf]
