# Supplementary figures and images for: RNA-binding protein YebC enhances translation of proline-rich amino acid stretches in bacteria
Source: Nat Commun. 2025 Jul 7;16:6262. doi: 10.1038/s41467-025-60687-4 (PMC12234827; doi:10.1038/s41467-025-60687-4)

Figure 2B: PNK assay

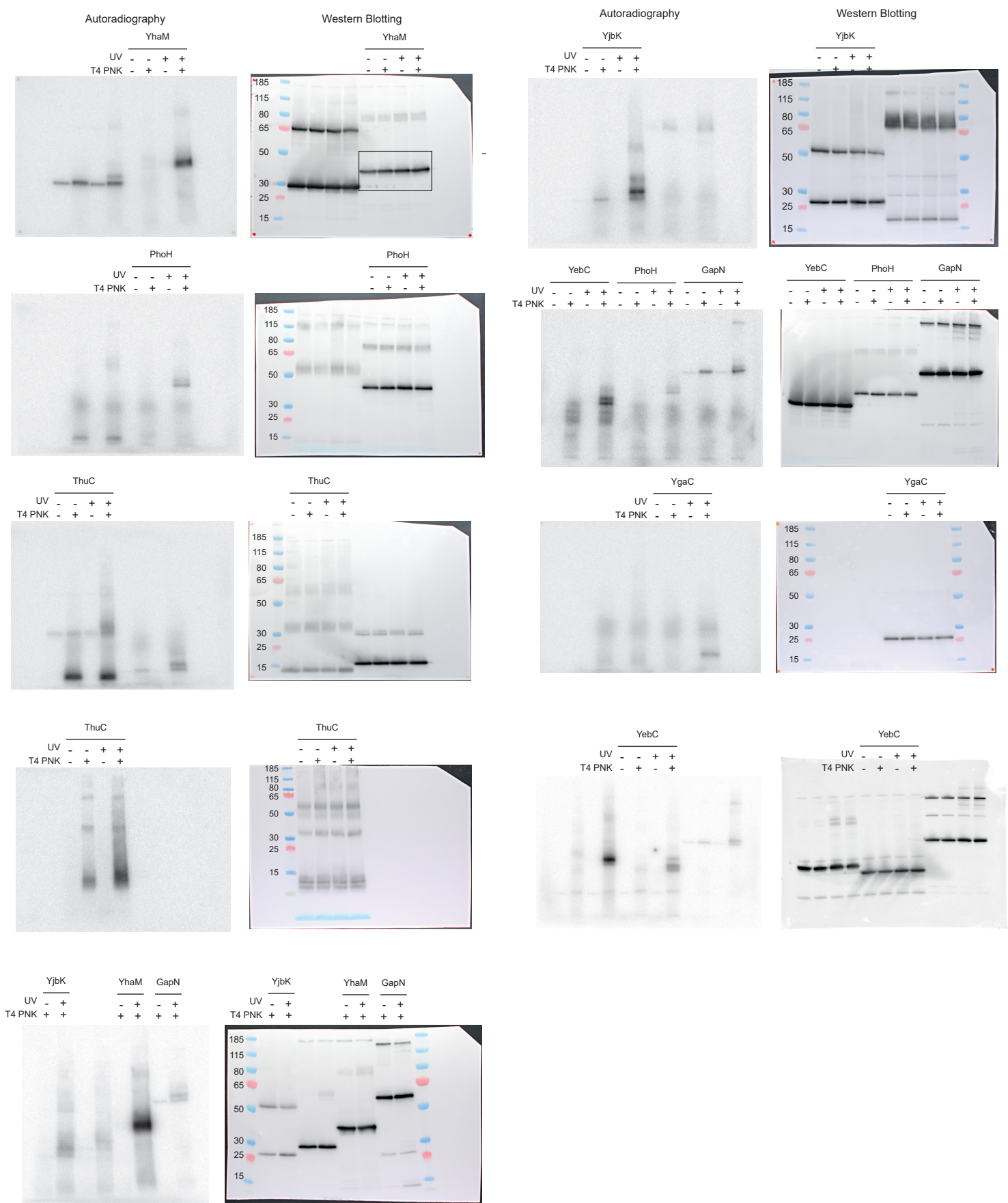

Supplement: Supplementary file 12 — Source Data [file 41467_2025_60687_MOESM12_ESM.zip › 250519_Source_Data/Fig_2B_PNK assay.pdf]

Figure 3A: SpeB translation reporters

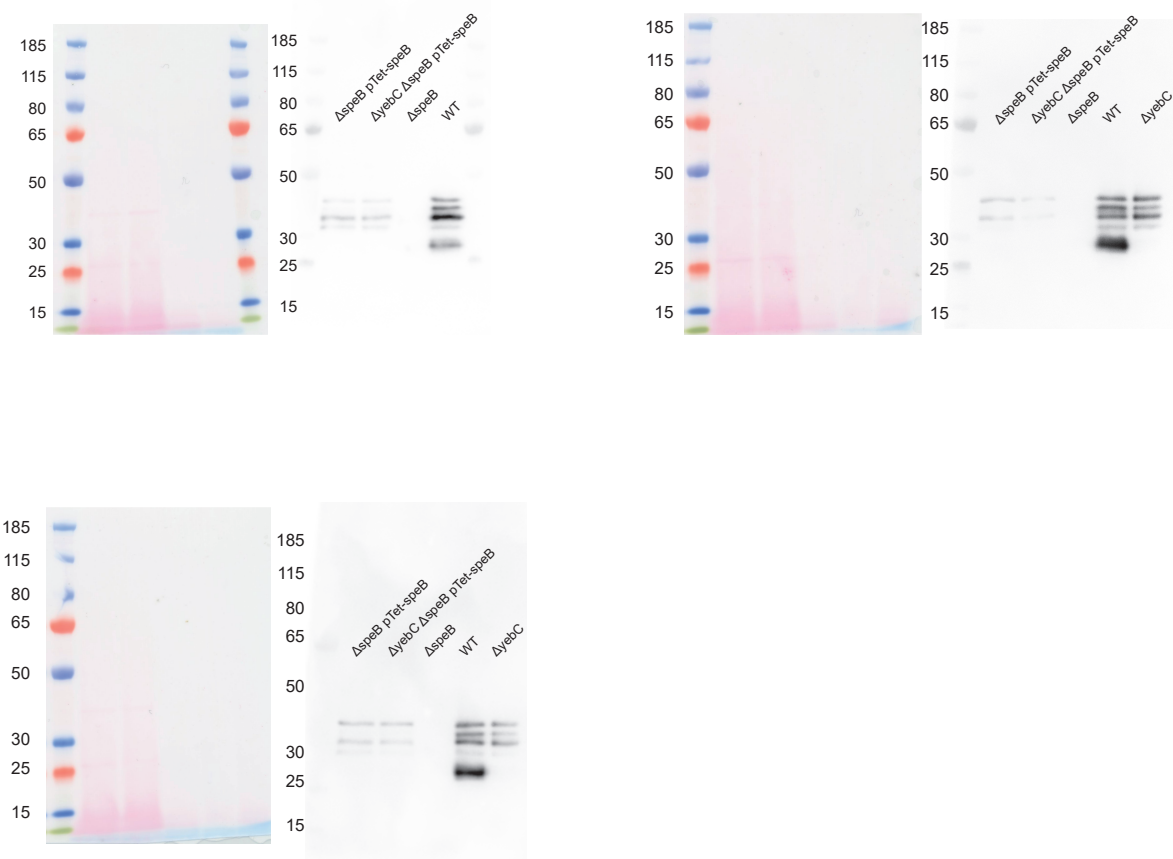

Supplement: Supplementary file 12 — Source Data [file 41467_2025_60687_MOESM12_ESM.zip › 250519_Source_Data/Fig_3B_SpeB_translation_reporters.pdf]

**Figure 3B: SpeB transcription reporters**

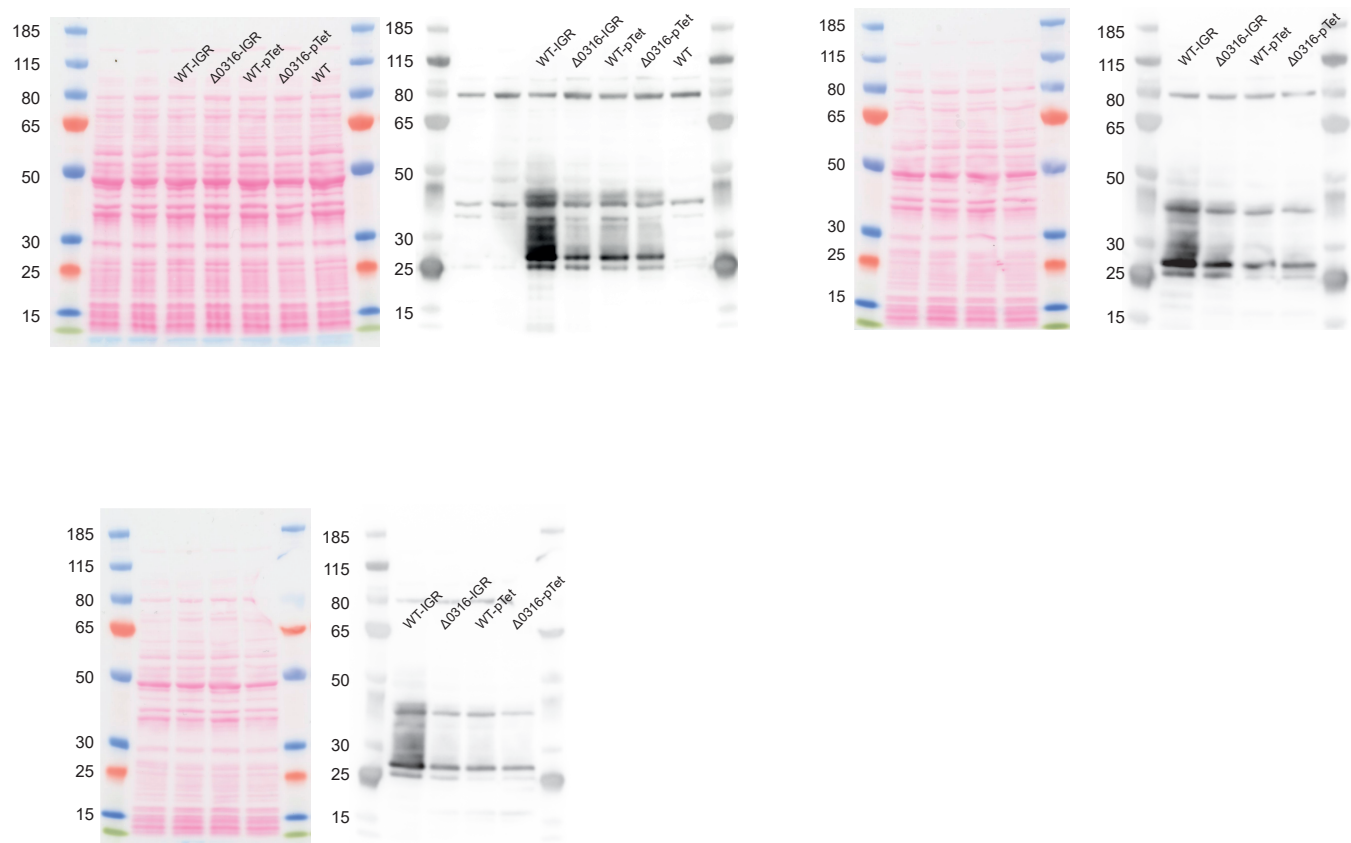

Supplement: Supplementary file 12 — Source Data [file 41467_2025_60687_MOESM12_ESM.zip › 250519_Source_Data/Fig_3C_SpeB_transcription_reporters.pdf]

**Figure 4B: Expression of YebC mutant versions**

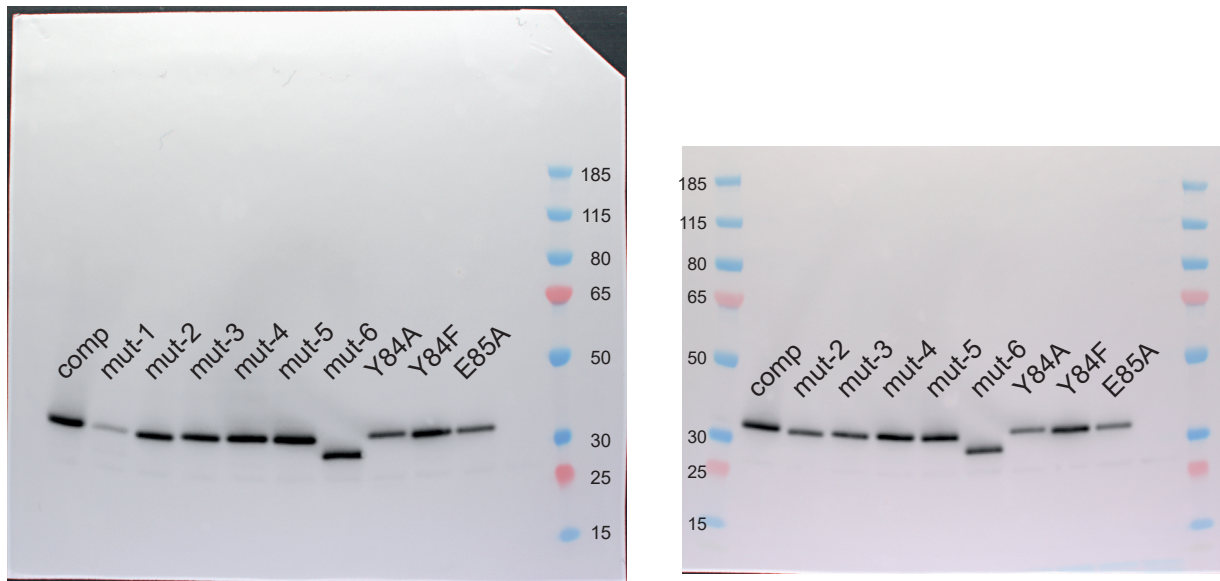

Supplement: Supplementary file 12 — Source Data [file 41467_2025_60687_MOESM12_ESM.zip › 250519_Source_Data/Fig_4B_WB_YebC_yebC_mutants_M1-Y85E.pdf]

**Figure 4C: SpeB expression**

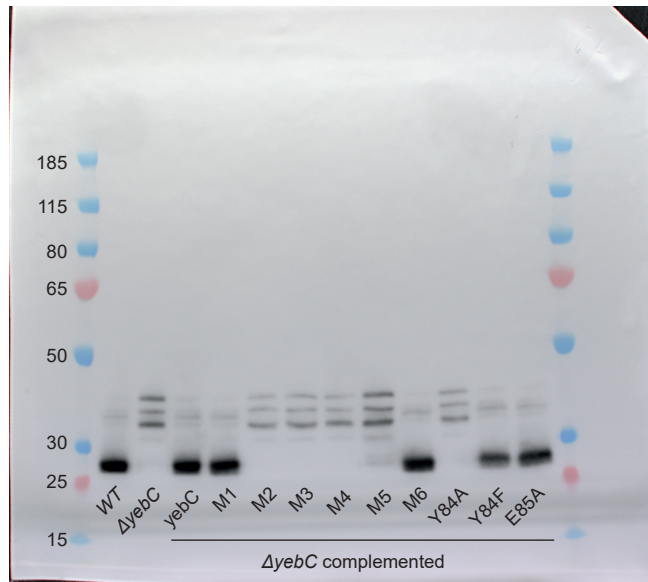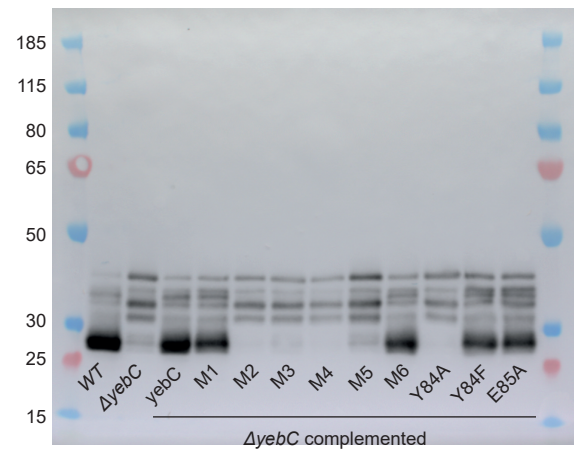

Supplement: Supplementary file 12 — Source Data [file 41467_2025_60687_MOESM12_ESM.zip › 250519_Source_Data/Fig_4C_SpeB_yebC_mutants_M1-Y85E.pdf]

Figure 4D: OOPS for YebC mutants M2 and Y84A

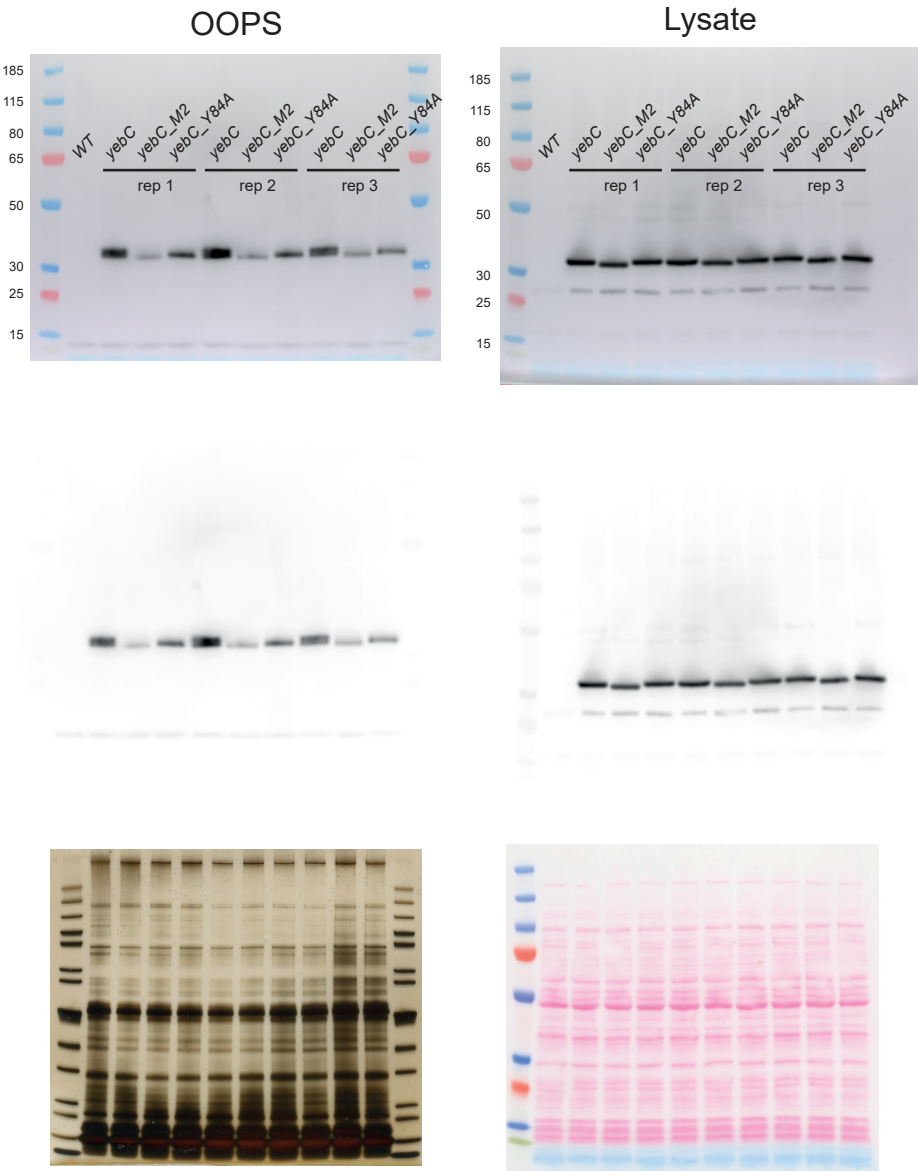

Supplement: Supplementary file 12 — Source Data [file 41467_2025_60687_MOESM12_ESM.zip › 250519_Source_Data/Fig_4D_WB_OOPS_yebC_M2_Y84A.pdf]

**Figure 7A: *in vivo* polyproline reporters**

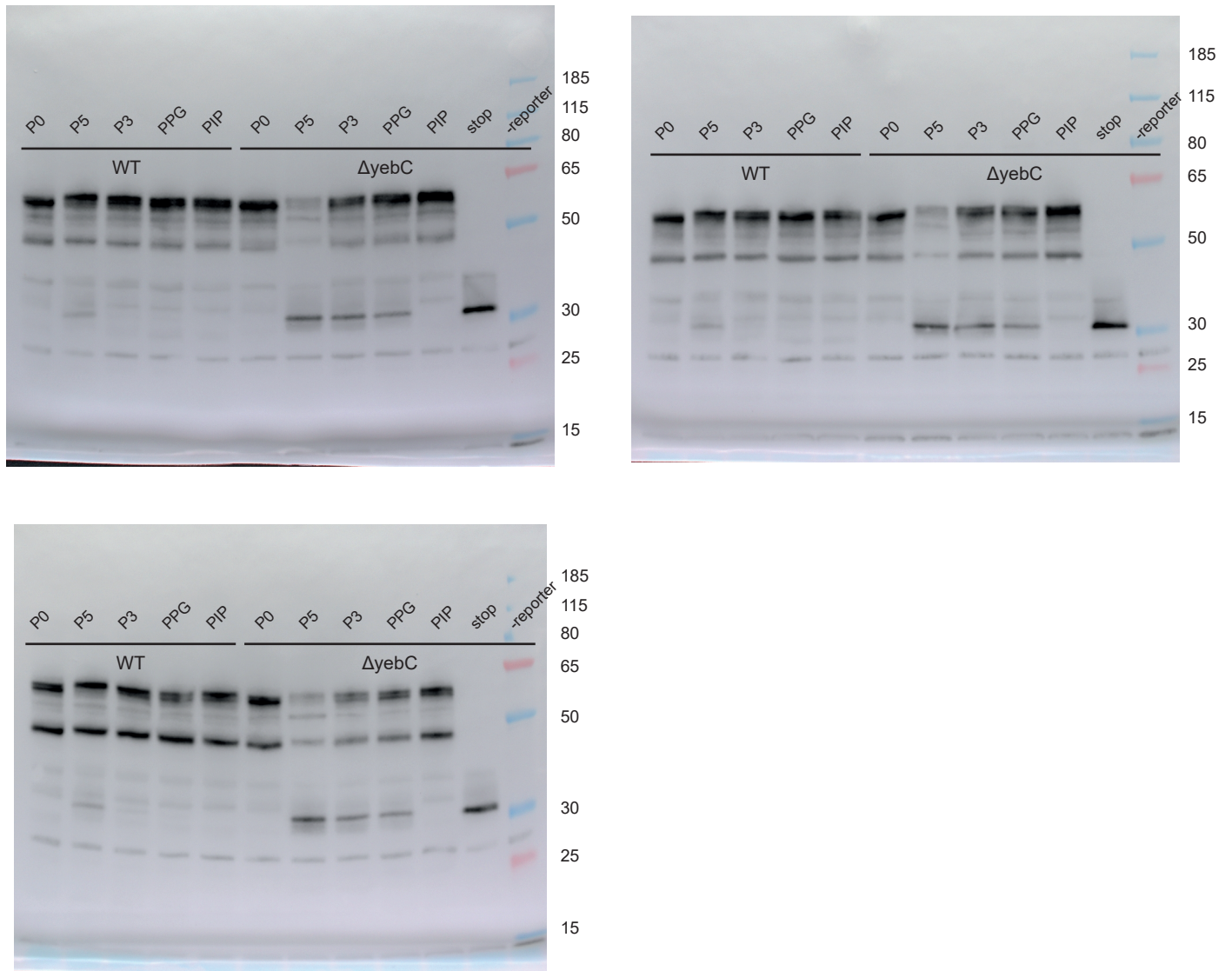

Supplement: Supplementary file 12 — Source Data [file 41467_2025_60687_MOESM12_ESM.zip › 250519_Source_Data/Fig_7A_WB_in_vivo_polyproline_reporters.pdf]

Figure 8A: in vitro polyproline reporters

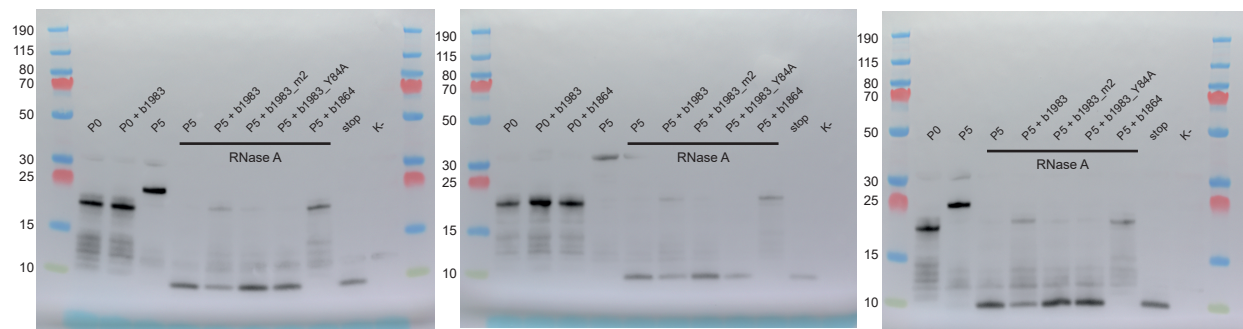

Supplement: Supplementary file 12 — Source Data [file 41467_2025_60687_MOESM12_ESM.zip › 250519_Source_Data/Fig_8A_WB_in_vitro_polyproline_reporters.pdf]

Supplementary figure 1A: UV titration for OOPS

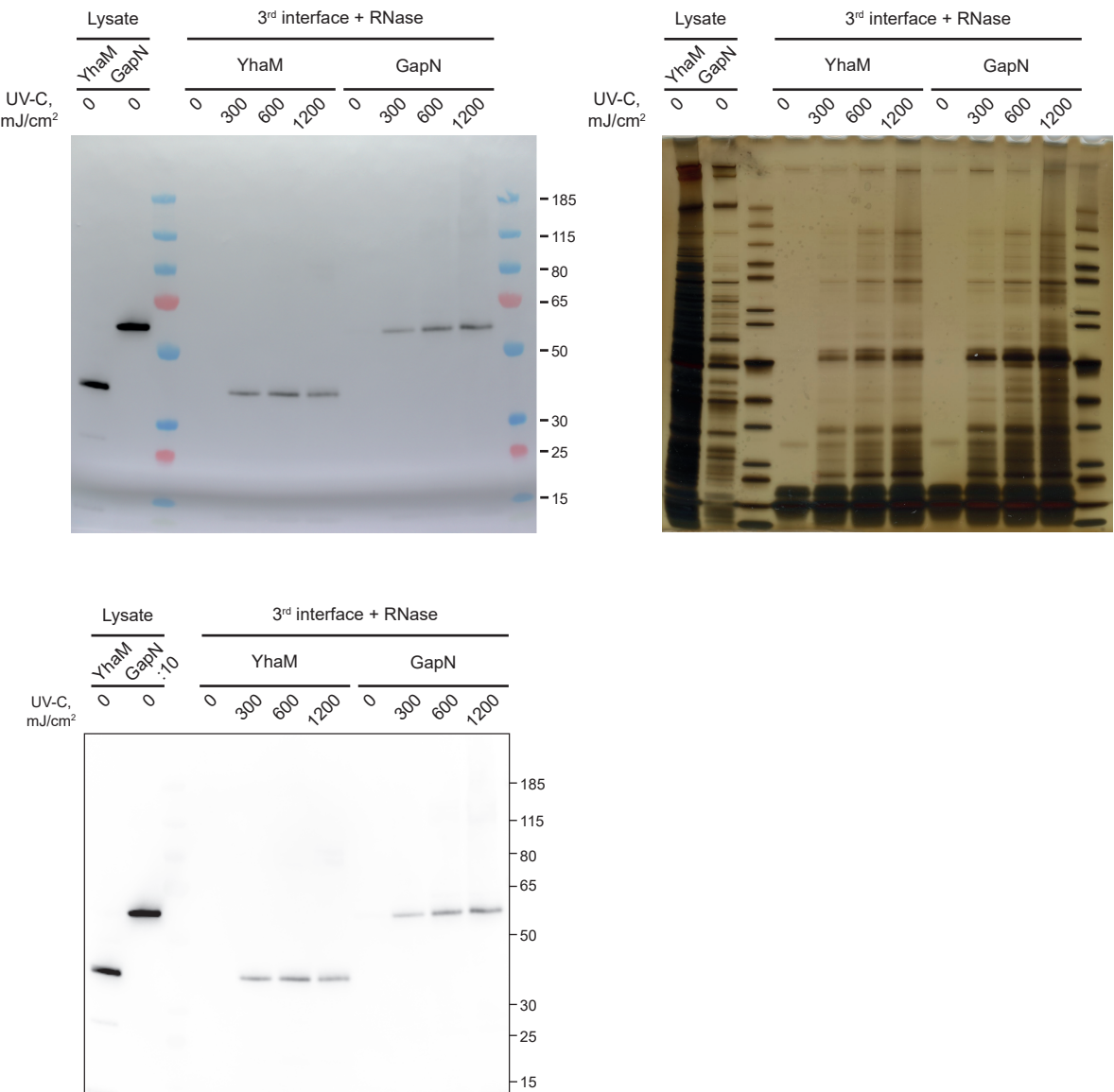

Supplement: Supplementary file 12 — Source Data [file 41467_2025_60687_MOESM12_ESM.zip › 250519_Source_Data/Fig_S1_OOPS_UV_titr.pdf]

**Supplementary figure 5A: Expression of YebC in THY and CDM**

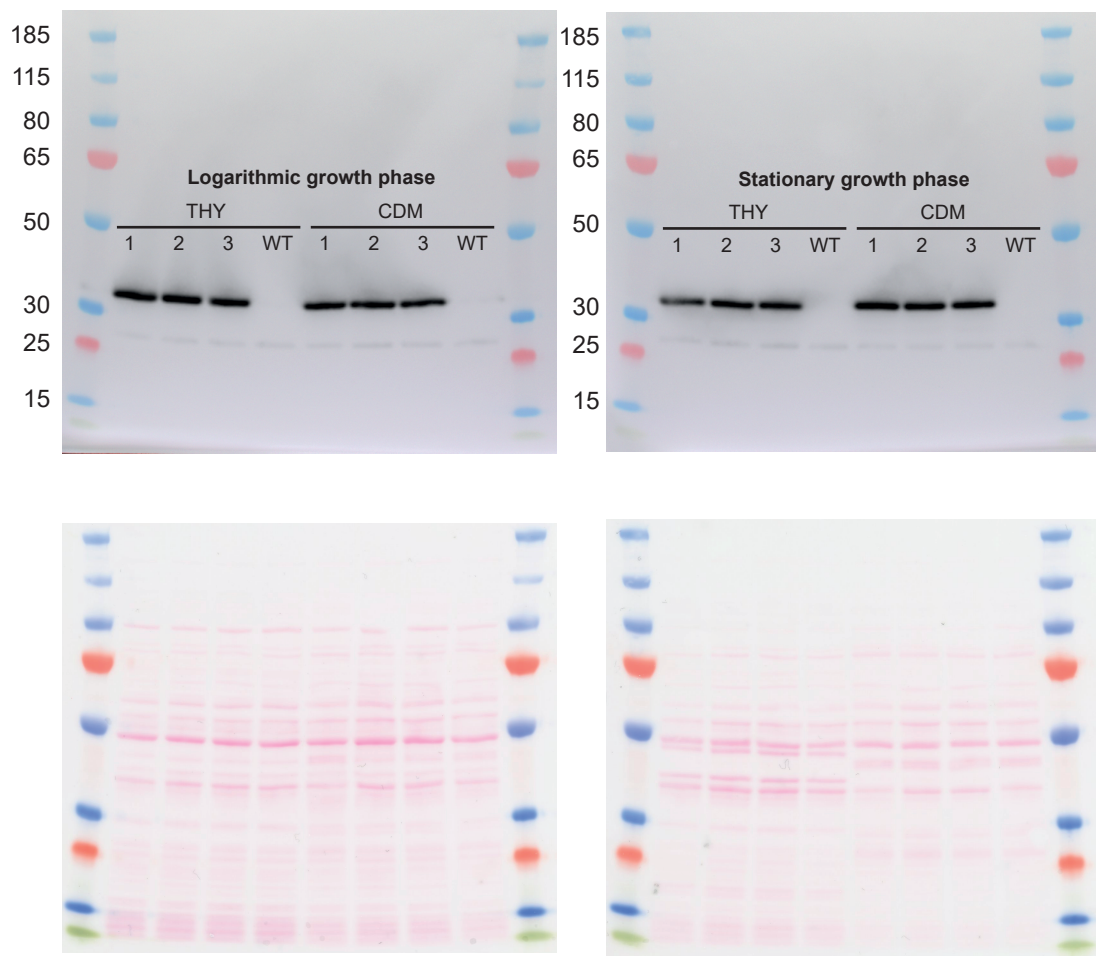

Supplement: Supplementary file 12 — Source Data [file 41467_2025_60687_MOESM12_ESM.zip › 250519_Source_Data/Fig_S5A_YebC_in_THY_CDM.pdf]

Figure S8: RNase titration for iCLIP for yebC

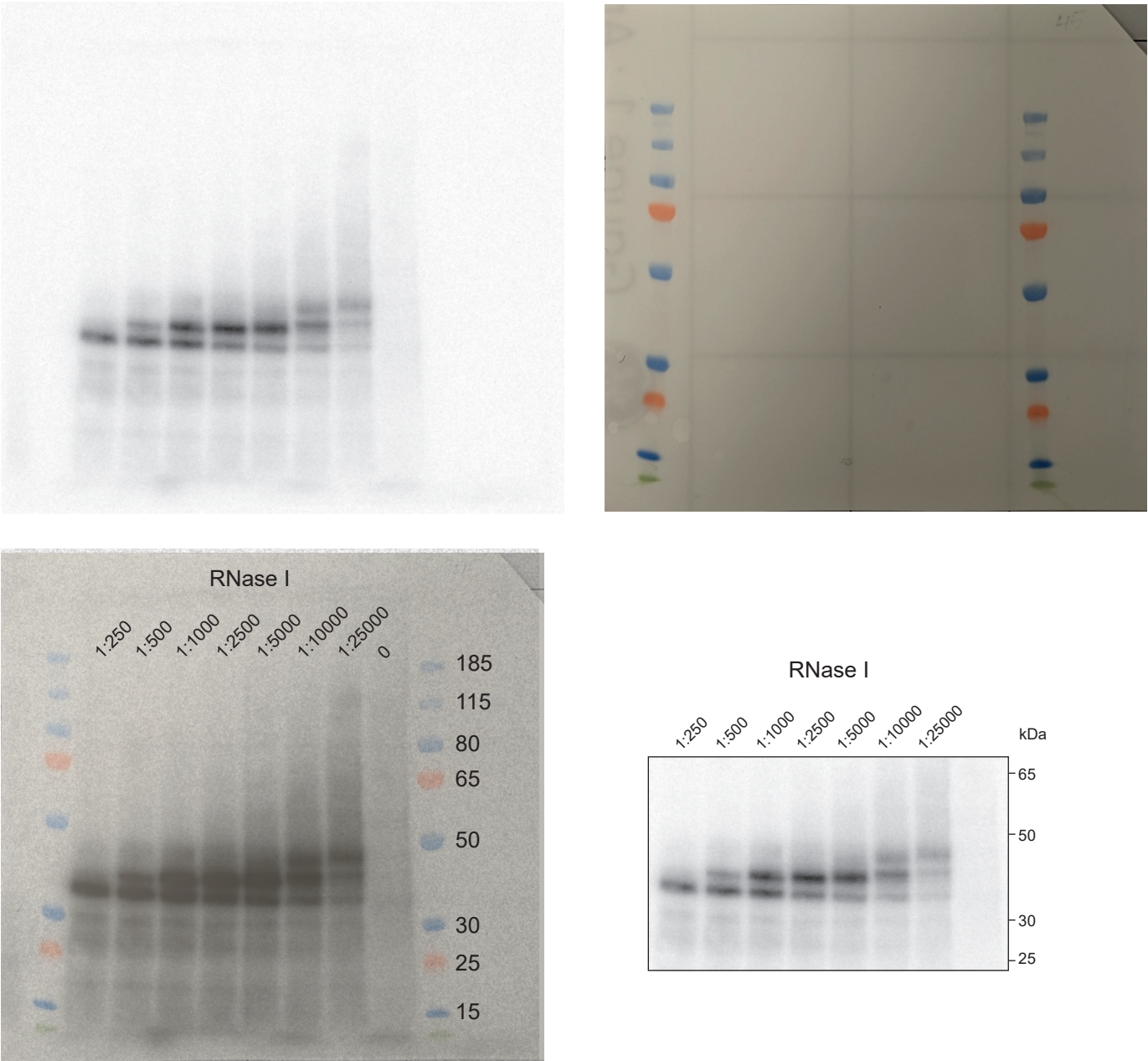

Supplement: Supplementary file 12 — Source Data [file 41467_2025_60687_MOESM12_ESM.zip › 250519_Source_Data/Fig_S8_phosphphoimager_iCLIP_yebC_RNase_titration.pdf]
